# Cardiovascular biomarkers in feline hypertrophic cardiomyopathy phenotype: evidence from the last decade

**DOI:** 10.1007/s11259-026-11408-9

**Published:** 2026-07-17

**Authors:** Felipe Gaia de Sousa, Ruthnea Aparecida Lázaro Muzzi, Fernanda do Carmo Magalhães, Roberto Baracat de Araújo, Rafael Resende Faleiros, Suzane Lilian Beier

**Affiliations:** 1 Department of Veterinary Clinic and Surgery, School of Veterinary Medicine, Federal University of Minas Gerais - UFMG, 6627 Antônio Carlos Av, Pampulha, Belo Horizonte, Minas Gerais 31270-901 Brazil; 2 VETHEART – Cardiovascular Physiology and Veterinary Cardiology Research Group, Belo Horizonte, Minas Gerais Brazil; 3 INCT Nanobiofar – National Institute of Science and Technology in Nanobiopharmaceutics, Belo Horizonte, Minas Gerais Brazil; 4 Department of Veterinary Medicine, Faculty of Animal Science and Veterinary Medicine, Federal University of Lavras - FZMV/UFLA, Lavras, Minas Gerais Brazil; 5 Department of Preventive Veterinary Medicine, Veterinary School, Federal University of Minas Gerais – UFMG, Belo Horizonte, Minas Gerais Brazil

**Keywords:** Biological markers, Cardiomyopathies, Cats, Diagnostic, Prognostic value

## Abstract

The hypertrophic cardiomyopathy (HCM) phenotype is the most common feline cardiovascular condition, characterized by myocardial hypertrophy and variable hemodynamic changes, with diagnosis primarily based on echocardiography. However, cardiovascular biomarkers have gained increasing importance in the diagnostic assessment of the HCM phenotype. This systematic review aimed to evaluate the utility of cardiovascular-related biomarkers in the diagnosis of HCM in cats, in order to determine their clinical potential and utility. A systematic search was conducted in PubMed, Scopus, and Web of Science over a last decade (January 2015 – June 2025). Study selection was performed by a single reviewer, and risk-of-bias assessment by two reviewers using the Joanna Briggs Institute (JBI) tools. Studies providing relevant data on the use of cardiovascular biomarkers in cats with the HCM phenotype were included. Data regarding study design, population characteristics, biomarker type, and clinical associations were extracted and analyzed. Out of 220 initially screened studies, 63 met the inclusion criteria and the majority were considered of high methodological quality with low risk of bias. The most frequently reported biomarkers were N-terminal pro–B-type natriuretic peptide (NT-proBNP) and cardiac troponin I (cTnI), both commonly associated with clinical status, echocardiographic findings, and prognostic risk. Other emerging biomarkers, including molecular and genetic markers related to myocardial remodeling and fibrosis, demonstrated potential clinical utility, although further investigation is still required. Overall, despite robust scientific evidence, biomarkers play an adjunctive role in the evaluation of the feline HCM phenotype. Echocardiography remains the gold standard for definitive diagnosis.

**Supplementary Information:**

The online version contains supplementary material available at https://doi.org/10.1007/s11259-026-11408-9.

## Introduction

**Fig. 1:**
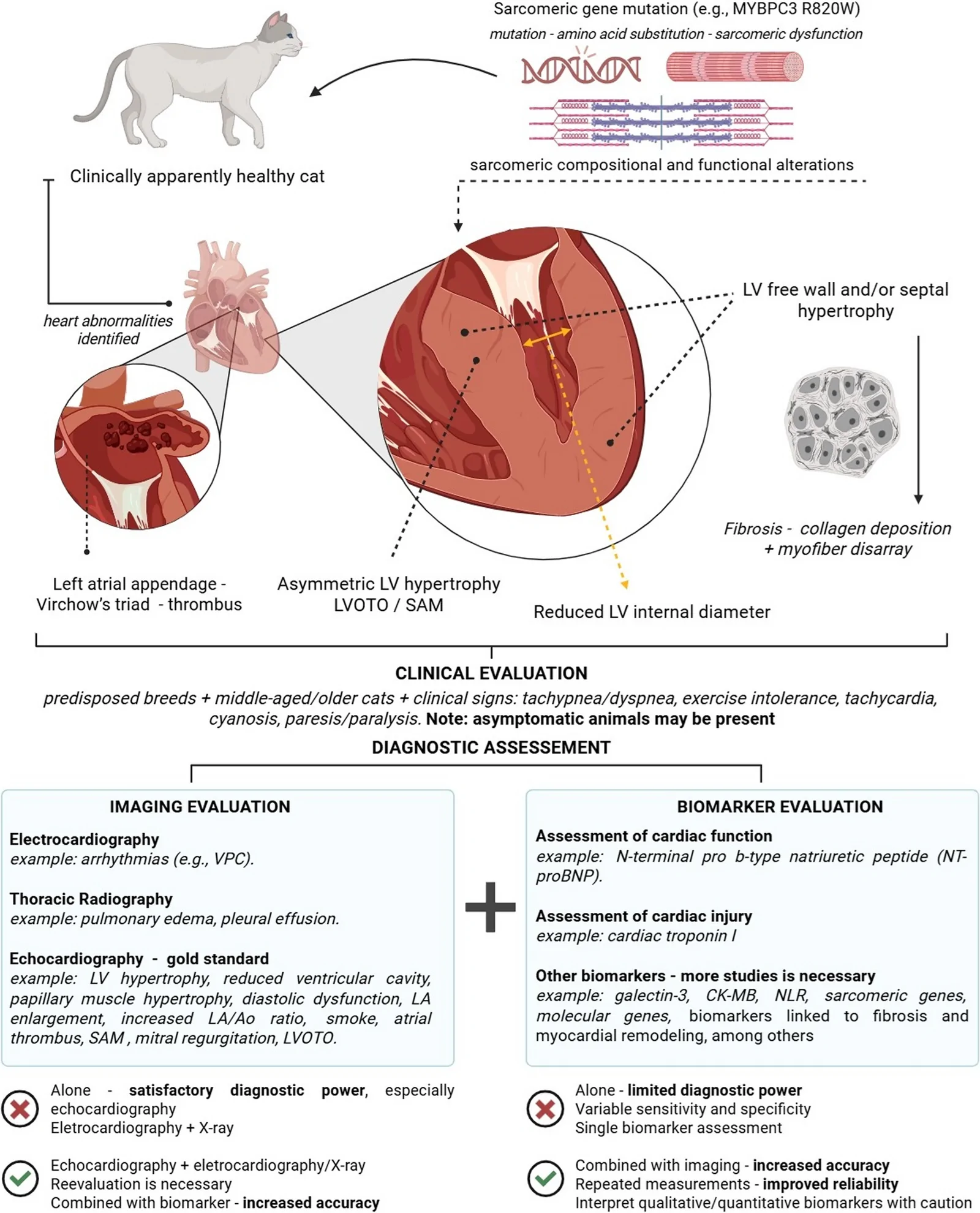
Pathophysiology and integrated diagnostic approach of the feline HCM phenotype. Created by the authors with BioRender (https://www.biorender.com/)

The feline hypertrophic cardiomyopathy (HCM) phenotype is characterized by concentric hypertrophy of the left ventricle (LV), reduced LV internal diameter, and left atrial dilation. In some cases, it may also be associated with systolic anterior motion (SAM) of the mitral valve and left ventricular outflow tract obstruction (LVOTO) due to segmental hypertrophy (Fig. 1) (Payne et al. 2010, 2013, 2015; Luis Fuentes and Wilkie 2017; Fox et al. 2018, 2019; Luis Fuentes et al. 2020; Novo Matos et al. 2022, 2023a; Novo Matos and Payne 2023; Seo et al. 2024b, 2025; de Sousa et al. 2025; Gaia de Sousa et al. 2025). The phenotype may be classified as primary HCM (genetic origin) or secondary (HCM phenocopies) (Meurs et al. 2005, 2007; Luis Fuentes et al. 2020; Janus et al. 2023; de Sousa et al. 2025; Kaplan et al. 2025). It is the most commonly diagnosed cardiovascular phenotype in middle-aged to older cats, with recognized breed predispositions linked to genetic factors (Meurs et al. 2005, 2007; Fox et al. 2018, 2019; Luis Fuentes et al. 2020; Kaplan et al. 2025).

Affected cats are categorized into five stages according to the American College of Veterinary Internal Medicine (ACVIM) consensus guidelines: stage A (at risk), B1 and B2 (subclinical), C (clinical), and D (refractory) (Luis Fuentes et al. 2020). The genetic basis of the phenotype has been associated with sarcomeric mutations, particularly in the MYBPC3 gene, leading to alterations in amino acid structure and impaired sarcomeric protein production (Fig. 1) (Meurs et al. 2005, 2007; Borgeat et al. 2015b; Kaplan et al. 2025). Consequently, sarcomere function is disrupted, and myocardial fibers are progressively replaced by disorganized fibrous connective tissue. This abnormal arrangement of myocardial fibers interspersed with collagen contributes to myocardial fibrosis and impaired cardiac relaxation (Borgeat et al. 2015b). As a result, left ventricular diastolic function is compromised, leading to significant hemodynamic alterations.

In the early stages, cats may compensate effectively; however, depending on the severity of hypertrophy and compensatory tachycardia, the condition may progressively worsen, leading to further cardiac dysfunction. Clinical manifestations are therefore variable and stage-dependent (Luis Fuentes et al. 2020; Kittleson and Côté 2021a; de Sousa et al. 2025; Gaia de Sousa et al. 2025). Cats with advanced HCM are more likely to exhibit overt clinical signs, particularly cardiovascular complications such as congestive heart failure (CHF) and/or arterial thromboembolism (ATE) (Payne et al. 2015; Luis Fuentes et al. 2020; Kittleson and Côté 2021a, b; Novo Matos et al. 2022; Guillaumin 2024; de Sousa et al. 2025; Sousa et al. 2026; Seo et al. 2025). Notably, a substantial proportion of cats remain subclinical, which complicates early detection and may negatively impact the assessment of therapeutic efficacy (Luis Fuentes and Wilkie 2017; van Hoek et al. 2020a; Luis Fuentes et al. 2020; de Sousa et al. 2025; Carter et al. 2026).

The diagnosis of the HCM phenotype is based on the integration of clinical, laboratory, and imaging findings, with conventional and advanced echocardiography (Fig. 1) (Luis Fuentes et al. 2020; Kittleson and Côté 2021a; Fries 2023; de Sousa et al. 2025; Gaia de Sousa et al. 2025). Although no single clinical or laboratory finding is pathognomonic for HCM, certain tools may provide valuable information, including cardiac biomarkers. Biomarkers are measurable substances in blood or plasma that reflect physiological or pathological processes, particularly those related to cardiovascular function or injury (Ahmad et al. 2023; Netala et al. 2025). They can be broadly classified as markers of myocardial function or injury, such as N-terminal pro–B-type natriuretic peptide (NT-proBNP) and cardiac troponin I (cTnI) (Gaia de Sousa et al. 2025; Netala et al. 2025).

Biomarker release into the circulation depends on the underlying pathophysiological process. For example, myocardial injury is associated with increased circulating cTnI levels (Fig. 1) (Langhorn et al. 2014; Hori et al. 2018; Hanås et al. 2022; Netala et al. 2025; Satomi et al. 2025), whereas myocardial stretch, such as that occurring during atrial remodeling, leads to elevated plasma NT-proBNP concentrations (Fig. 1) (Hsu et al. 2009; Machen et al. 2014; Pierce et al. 2017; Gaia de Sousa et al. 2025). In this context, biomarkers may serve as useful tools in cardiovascular assessment. However, despite their availability, current evidence supports their role primarily as complementary tools in the diagnosis of HCM, with echocardiography remaining essential for definitive confirmation of the phenotype. Additionally, factors such as cost and availability may limit the widespread use of biomarkers in certain regions (de Sousa et al. 2025). Therefore, the aim of this systematic review was to evaluate the performance of cardiovascular biomarkers in the diagnosis of cats with the HCM phenotype.

## Materials and methods

### Study design

This study was designed as a systematic review (January 2015 – June 2026) aiming to evaluate the diagnostic performance of cardiovascular biomarkers in cats with hypertrophic cardiomyopathy. The systematic review was conducted in accordance with the Preferred Reporting Items for Systematic Reviews and Meta-Analyses (PRISMA) guidelines, following the PRISMA 2020 flow diagram. This approach included comprehensive searches of electronic databases, study records, and additional sources. As this study did not involve the use of experimental or non-experimental animals, but rather consisted of a systematic review of previously published prospective and retrospective studies, based on predefined search, screening, and analytical criteria, ethical approval from an institutional animal care and use committee was not required.

### Search strategy, data characterization and collection

**Fig. 2:**
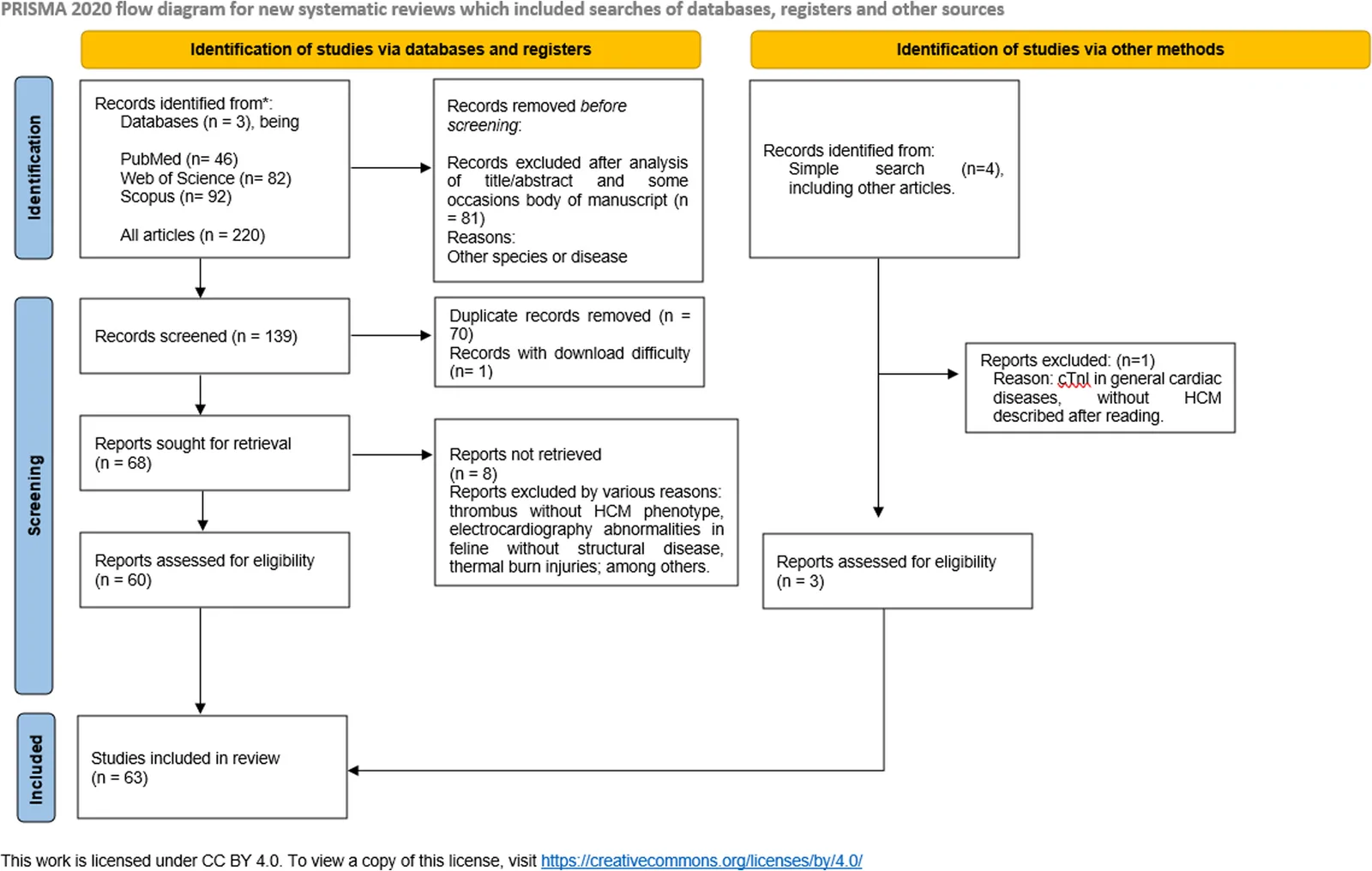
PRISMA 2020 flowchart highlighting the entire study screening process using defined databases, registries, and other sources. Source: https://www.prisma-statement.org/.

The search strategy was initially based on the development of a structured search equation to facilitate data retrieval and to achieve the primary objective of identifying evidence regarding the utility of cardiovascular-related biomarkers in cats with the HCM phenotype. The evidence search was conducted using the following descriptors: (“Cardiac Biomarkers” OR “Biological Markers” OR “cardiac biomarker*” OR “cardiac troponin” OR troponin OR “NT-proBNP” OR BNP) AND (“Cardiomyopathy, Hypertrophic” OR “hypertrophic cardiomyopathy” OR HCM OR “cardiac phenotype”) AND (“Cats” OR cat OR cats OR feline OR felines). The search equation was applied to the search fields of three different electronic databases, namely: PubMed (https://pubmed.ncbi.nlm.nih.gov/), Web of Science (https://webofknowledge.com/) e Scopus (https://www.scopus.com/search/). At the time of this study, no systematic review had specifically evaluated the impact of available evidence regarding the use of cardiovascular-related biomarkers in feline patients with the HCM phenotype. Therefore, primary studies were collected, excluding review articles. No conference abstracts were identified during the systematic search. Only studies published in the English language were included. When relevant and not captured by the initial search strategy, additional studies were identified through manual screening of reference lists and included for full-text assessment. Reference lists of included studies were not systematically searched. The detailed processes of screening, selection, and data analysis are presented in the PRISMA 2020 flow diagram (Fig. 2).

### Screening and review data

A total of 220 articles were initially identified across the three databases, including 46 from PubMed, 82 from Web of Science, and 92 from Scopus (Fig. 2). The screening process followed predefined search criteria, beginning with the evaluation of titles and abstracts, followed by the removal of duplicate records. Documents classified as review articles, those involving human subjects or species other than felines, and studies not aligned with the primary objective were excluded. The initial selection of manuscripts was strongly supported by visual and dynamic assessment, as well as preliminary evaluation of titles and abstracts. In cases where studies addressed the HCM phenotype but did not explicitly mention biomarkers in the abstract, a rapid preliminary full-text screening was performed. If relevant data on the topic were identified, the studies were included. Subsequently, all selected articles underwent a comprehensive and detailed full-text review. Studies that did not meet the inclusion criteria regarding the use of biomarkers in the diagnosis of the HCM phenotype were excluded at this stage. The assessment of data reliability and the potential risk of bias also contributed to the final selection of studies. As part of the screening and document analysis process, several articles were excluded for various reasons, which are detailed in the “Exclusion of studies” section (Fig. 2).

### Eligibility and analysis

Following the selection and analysis of articles (January 2015 – June 2026), all studies reporting data on the use of biomarkers as diagnostic tools in this cardiac condition underwent eligibility assessment, and relevant evidence was subsequently extracted for analysis (Fig. 2).

### Data extraction and synthesis

After the screening, selection, and analysis phases, all included studies were fully reviewed, and data were systematically extracted and tabulated in a Microsoft^®^ Excel^®^ spreadsheet (Microsoft 365 MSO, Version 2508, Build 16.0.19127.20082). The extracted data for each study were organized into predefined fields, including publication year, study design, animal characteristics (number, sex, and age), HCM stage, biomarker type and cut-off values (when available), presence or absence of clinical signs, and corresponding references (Online Resource 1). This structured tabulation enabled the evaluation of the total number of studies and animals included, the study period, the presence of clinical manifestations, the diagnostic utility of biomarkers, and their prognostic relevance (Fig. 2). Data were synthesized descriptively according to biomarker type, study design, clinical classification, and reported diagnostic and prognostic outcomes. A quantitative meta-analysis was not performed because the included studies exhibited substantial heterogeneity in terms of study design, clinical classifications, biomarker cut-off values, study populations, and reported outcomes, thereby precluding a meaningful quantitative synthesis.

### Analyses of quality evidence

The quality of evidence was assessed in accordance with the Joanna Briggs Institute (JBI) guidelines (https://jbi.global/), allowing each study to be evaluated based on the characteristics of its evidence. A specific critical appraisal checklist was applied to all included studies, taking into account their respective study designs (observational, molecular, diagnostic, case report, and experimental). The evidence was classified and tabulated according to the description of findings, risk of bias, clinical applicability, and consistency of results. Quality classification was based on a percentage score, calculated as the sum of the scores assigned by two independent reviewers, divided by the maximum possible score and multiplied by 100. Studies with scores > 70% were considered high quality, those scoring between 50 and 70% were classified as moderate quality, and those with scores < 50% were considered low quality.

## Risk of bias assessment

The methodological quality and risk of bias of the included studies were independently assessed by two reviewers using a standardized tool appropriate for the study design. Discrepancies were resolved by consensus. The assessment considered domains such as selection bias, measurement bias, confounding, and reporting bias. Overall, the included studies showed [low/moderate/high] risk of bias, mainly due to limitations in study design, incomplete reporting, and lack of control for confounding factors.

### Exclusion of studies

Several studies were excluded following the completion of the screening, data collection, and analysis processes. The reasons for exclusion included studies involving other species (such as dogs and kangaroos), review articles, studies evaluating biomarkers in general cardiac diseases without specific distinction for the HCM phenotype, and those related to different conditions (e.g., lymphoma), as well as difficulties in accessing full-text articles (Fig. 2).

## Results

### Identification and selection of relevant articles

Following the identification of 220 records across the selected databases and the manual inclusion of three additional studies, a total of 63 articles were ultimately included in the final analysis (Fig. 2). The different databases yielded varying numbers of records based on the automated search strategy using the previously described search equation. Of the 220 initially identified documents, 81 were excluded after preliminary screening of titles and/or abstracts. In addition, 70 duplicate articles were removed because the same studies were indexed across multiple databases, and one article was excluded due to download difficulties (Fig. 2). The remaining 68 articles identified through the automated search strategy after the initial screening underwent full-text assessment, resulting in the exclusion of eight studies. These exclusions were based on the absence of a hypertrophic phenotype despite cardiac alterations, studies involving clinical conditions such as post-burn injury, and cases of transient myocardial thickening. At the end of the automated search process, 60 studies were deemed eligible, consisting predominantly of original research articles and a smaller number of case reports. To enhance the robustness of the evidence synthesis, three additional studies not captured by the initial search equation were manually included based on their relevance (Tantitamtaworn et al. 2024; Joshua et al. 2025; Abdelhaleem et al. 2025). Thus, after the complete process of systematic screening and application of eligibility criteria, a total of 63 studies were considered suitable for detailed analysis and critical appraisal (Fig. 2).

### Study characteristics after data extraction

Data extraction was performed in accordance with the predefined inclusion and exclusion criteria, as well as the screening and eligibility processes applied to studies published between 2015 and 2026. The extracted data are presented in the Online Resource 1. The data extraction table was structured using Microsoft^®^ Excel^®^ for Microsoft 365 MSO (Version 2508, Build 16.0.19127.20082) to include the variables of interest, namely: year and study design, animal characteristics, HCM phenotype stage, biomarkers, main findings, and references. Particularly in the “animals” field, detailed information was collected, including number of animals, sex, age range, breed, proportion of males and females, group allocation, and, in the case of case reports, the clinical signs presented (Online Resource 1).

Among the 63 studies included, most were published in 2015 (*n* = 4), 2016 (*n* = 2), 2017 (*n* = 4), 2018 (*n* = 5), 2019 (*n* = 3), 2020 (*n* = 12), followed by 2021 (*n* = 5), 2022 (*n* = 3), 2023 (*n* = 8), 2024 (*n* = 7), 2025 (*n* = 6) and 2026 (*n* = 4) (Online Resource 1). Approximately 35 studies were observational in design, predominantly retrospective, while only seven were case reports. In 58.70% of the studies, animals were stratified into groups, typically controls and diseased cats (with or without associated CHF and/or ATE). Affected cats generally exhibited higher mean or median age values, and sex distribution was more frequently in intact or neutered males. In some studies, animals commonly presented with clinical signs of CHF and/or ATE. Notably, a substantial proportion of studies did not classify affected cats according to the ACVIM HCM staging system (Online Resource 1). Instead, diagnosis was often based solely on echocardiographic criteria, including increased left ventricular free wall thickness (LVFWd) or interventricular septal thickness (IVSd) (> 6 mm), left atrial enlargement (LAE), and increased left atrium-to-aorta ratio (LA/Ao).

Regarding biomarkers, approximately 74.60% of the studies reported data on NT-proBNP/cTnI, and 41.26% investigated other biomarkers, including those associated with fibrosis, myocardial remodeling, inflammation, serological markers, and molecular pathways (Online Resource 1). Only 19.04% studies reported diagnostic cut-off values; in the remaining studies, reference values were consistently provided. In some cases, results were obtained using qualitative methods, although the majority of studies reported quantitative biomarker concentrations. Overall, serial measurements of biomarkers were rarely reported, with most studies relying on single time-point assessments without longitudinal monitoring.

The timing of biomarker evaluation emerged as an important consideration, as these markers were most commonly used in conjunction with diagnostic workups rather than as standalone tools. Although biomarkers were frequently highlighted as providing valuable information, particularly in screening contexts, most studies emphasized the role of echocardiography. In many cases, biomarker data were correlated with imaging findings, especially echocardiographic parameters. Recent studies have also highlighted the promising role of molecular biomarkers in the diagnosis and monitoring of the feline HCM phenotype, particularly those related to troponin-associated mutations, microRNAs (miRNAs), and gene regulation, identifying them as potential diagnostic candidates. The included studies demonstrated that biomarkers offer diagnostic benefits, particularly when used in combination with other diagnostic modalities (Online Resource 1).

### Quality of evidence and risk of bias

The methodological quality assessment of all included studies demonstrated a predominance of high-quality evidence (Table 1). Of the 63 studies included in this review, 47 (74.60%) were classified as high quality (low risk of bias), 9 (14.29%) as moderate quality (moderate risk of bias), and 7 (11.11%) as low quality (high risk of bias). This distribution indicates that the majority of studies included in this review presented consistent methodological design and adequate reporting of diagnostic criteria, interventions, and evaluated outcomes. Studies classified as moderate quality showed specific limitations, primarily related to incomplete reporting of methodological aspects or lack of detailed information regarding follow-up and data analysis. All studies classified as low quality were case reports. Due to their inherent design, these studies exhibited more pronounced methodological limitations and less comprehensive reporting of procedures, which may increase the risk of bias and limit the robustness of the inferences, thereby affecting the interpretation of the results.

**Table 1:** Assessment of methodological quality and risk of bias of the studies included in the systematic review (January 2015– June 2026)

Year	Reference	Study type	Score (%)	Methodological quality	Risk of bias
2015	Solter et al. (2015)	Observational	75	High	Moderate
2015	Borgeat et al. (2015a)	Observational	75	High	Moderate
2015	Borgeat et al. (2015b)	Observational	70	Moderate	Moderate
2015	Freeman et al. (2015)*	Observational	70	Moderate	Moderate
2016	Roderick et al. (2017)	Observational	75	High	Moderate
2016	Langhorn et al. (2016)	Observational	75	High	Moderate
2016	Parzeniecka-Jaworska et al. (2016)	Observational	75	Moderate	Moderate
2017	Pierce et al. (2017)	Observational	87.5	High	Low
2018	Heishima et al. (2018)	Observational	81	Moderate to high	Moderate
2018	Langhorn et al. (2018)	Observational	75	High	Low
2018	Langhorn et al. (2018)	Observational	75	High	Moderate
2019	Loughran et al. (2019)	Observational	85	High	Low
2019	Bartoszuk et al. (2019)	Observational	85	High	Low
2020	Liu et al. (2020)*	Observational	75	High	Low
2020	Rohrbaugh et al. (2020)	Observational	75	High	Low
2020	Seo et al. (2020)*	Observational	75	High	Low
2020	van Hoek et al. (2020a)*	Observational	75	High	Low
2021	Fonfara et al. (2021)	Observational	75	High	Low
2021	O’Shaughnessy et al. (2021)*	Observational	75	High	Low
2021	Bakirel et al. (2021)	Observational	75	High	Low
2021	Ironside et al. (2021)	Observational	75	High	Low
2022	Fries et al. (2022)	Observational	75	High	Low
2022	Hanås et al. (2022)	Observational	75	High	Low
2022	Kaya and Bakırel (2022)	Observational	75	High	Low
2023	Li et al. (2023)*	Observational	75	High	Low
2023	Stack et al. (2023)*	Observational	75	High	Low
2023	Novo Matos et al. (2023b)	Observational	75	High	Low
2024	Tantitamtaworn et al. (2024)	Observational	75	High	Low
2024	Partington et al. (2024)*	Observational	75	High	Low
2024	Jiwaganont et al. (2024)	Observational	75	High	Low
2025	Neumann (2025)	Observational	75	High	Low
2025	Abdelhaleem et al. (2025)	Observational	75	High	Low
2025	Satomi et al. (2025)	Observational	75	High	Low
2026	Satomi et al. (2026)*	Observational	87.5	High	Low
2026	Oranges et al. (2026)	Observational	75	High	Low
2017	Hertzsch et al. (2019)	Diagnostic	87.5	High	Low
2017	Harris et al. (2017)*	Diagnostic	75	High	Moderate
2018	Ward et al. (2018)*	Diagnostic	87.5	High	Low
2018	Hori et al. (2018)	Diagnostic	80	High	Moderate
2020	Laudhittirut et al. (2020)	Diagnostic	75	High	Low
2020	Hanås et al. (2020)	Diagnostic	75	High	Low
2021	Lu et al. (2021)*	Diagnostic	75	High	Low
2026	Carter et al. (2026)*	Observational	75	High	Low
2026	Lidbury et al. (2026)	Observational	75	High	Low
2017	Messer et al. (2017)	Experimental	62.5	Moderate	Moderate
2020	Coleman et al. (2020)*	Experimental	75	High	Low
2020	van Hoek et al. (2020b)	Experimental	75	High	Low
2023	Kaplan et al. (2023)*	Experimental	75	High	Low
2023	Karp et al. (2023)*	Experimental	72	High	Low
2020	Sarcinella et al. (2020)	Case report	45	Low	High
2020	Hori et al. (2020)	Case report	45	Low	High
2020	Vezzosi et al. (2020)	Case report	45	Low	High
2023	Sidler et al. (2023)	Case report	45	Low	High
2023	Seddon et al. (2023)	Case report	45	Low	High
2024	Seo et al. (2024a)	Case report	45	Low	High
2025	Saponaro et al. (2025)	Case report	45	Low	High
2020	McNamara et al. (2020)*	Molecular	60	Moderate	Moderate
2023	Stern et al. (2023)*	Molecular	75	High	Low
2024	Guelfi et al. (2024)	Molecular	70	Moderate	Moderate
2024	Demeekul et al. (2024)	Molecular	73	High	Low
2024	Chong et al. (2024)	Molecular	70	Moderate	Moderate
2025	Ohlsson et al. (2025)	Molecular	68	Moderate	Moderate
2025	Joshua et al. (2025)	Molecular	72	High	Low

* Reported manufacturer-related funding, editorial roles, or potential conflicts of interest

## Discussion

The present study aimed to analyze the available evidence regarding the performance of cardiovascular biomarkers as diagnostic tools for the HCM phenotype. Studies included in this review demonstrated that cardiovascular biomarkers provide complementary support in the evaluation of cats with the HCM phenotype, although their diagnostic performance is insufficient for definitive diagnosis. These findings reinforce the role of biomarkers in the diagnostic workup of HCM, while confirming that echocardiography is used for phenotypic confirmation. Table 2 provides an overview of biomarkers with documented evidence, detailing their pathophysiological mechanisms, clinical uses, and prognostic relevance.

**Table 2:** Overview of cardiovascular biomarker types, mechanisms, and clinical relevance in feline HCM phenotype

Biomarker	Pathophysiological mechanism	Screening	Staging	Prognosis	Thromboembolic risk	
Wall stress	Myocardial stretch due to increased filling pressures and diastolic dysfunction (NT-proBNP, proANP, ANP)	High utility -cardiac vs. non-cardiac disease (NT-proBNP POC/SNAP); ANP and proANP less useful	Strong association with LAE, CHF, and structural severity	Associated with CHF, clinical progression, therapeutics, and mortality	Indirect association via atrial dilation and CHF	
Myocardial injury biomarkers	Cardiomyocyte injury from wall stress, ischemia, subendocardial hypoperfusion, and altered calcium sensitivity (cTnI)	Limited specificity in early disease but supportive diagnostic value	Increase with advanced HCM, LAE, CHF, and severe asymptomatic disease	Strong prognostic value in CHF, HOCM, decompensated HCM, and mortality	Indirect association with disease severity, LAE, and ATE	
Extracellular matrix remodeling markers	Collagen turnover and myocardial fibrosis (MMPs, TIMPs, CITP, PIIINP)	Limited utility	Potential association with early structural remodeling and LA enlargement	Fibrosis-related progression and collagen remodeling abnormalities	Possible link via atrial stiffness and blood stasis	
Inflammatory and immunomodulatory biomarkers	Systemic and myocardial inflammation, macrophage activation (IL-6, IL-18, TNF-α, SAA, Gal-3)	Limited utility	Associated with more severe phenotypes and CHF	Potential prognostic value (especially Gal-3 and IL-18)	Strong association with thrombosis through inflammation and endothelial activation	
Thrombosis and NETosis markers	Endothelial activation, hypercoagulability, and neutrophil extracellular traps (D-dimer, cfDNA, citH3)	Not used for screening	Increased in advanced disease and CHF	Strong prognostic value in ATE and mortality	Primary biomarker group directly linked to thromboembolism	
Metabolic hormonal axis	Growth signaling and myocardial hypertrophy modulation (IGF-1, insulin, glucose)	Limited utility	Associated with ventricular hypertrophy and myocardial growth	Possible role in structural progression	No established direct link	
Genetic biomarkers	Sarcomeric gene variants and regulatory RNAs influencing hypertrophy pathways (MYBPC3, TNNT2, miRNAs such as miR-122 and miR-370)	Potential future screening tool through genetic risk stratification	Differentiate primary vs. secondary cardiomyopathy and identify mutation-associated phenotypes	Experimental prognostic value	Indirect association through structural phenotype	
Hemodynamic and systemic indirect markers	Systemic congestion, organ hypoperfusion, secondary injury, renal dysfunction, and hematologic changes (creatinine, SDMA, RDW)	Not suitable for screening	Associated with CHF severity and systemic compromise	Associated with worse outcomes in CHF. RDW may help identify decompensated disease	Indirect association by CHF and circulatory dysfunction	

*ATE* Arterial thromboembolism, *cfDNA* Cell-free DNA, *CHF* Congestive heart failure, *citH3* Citrullinated histone H3, *CITP* C-terminal telopeptide of type I procollagen, *cTnI* Cardiac troponin I, *Gal-3* Galectin-3, *HCM* Hypertrophic cardiomyopathy, *HOCM* Hypertrophic obstructive cardiomyopathy, *IGF-1* Insulin-like growth factor 1, *IL* Interleukin, *LAE* Left atrial enlargement, *miRNAs* MicroRNAs, *MMP* Matrix metalloproteinase, *MYBPC3* Myosin-binding protein C3 gene, *NETosis* Neutrophil extracellular trap formation, *NT-proBNP* N-terminal pro–B-type natriuretic peptide, *PIIINP* N-terminal of type III procollagen, *POC* Point-of-care, *proANP *Pro–atrial natriuretic peptide, *RDW* Red cell distribution width, *SAA* Serum amyloid A, *SDMA* Symmetric dimethylarginine, *TIMP* Tissue inhibitor of metalloproteinases, *TNF-α* Tumor necrosis factor alpha

The assessment of methodological quality and risk of bias demonstrated a predominance of studies with low risk of bias, reinforcing the consistency of the evidence synthesized in this review (Table 1). The findings reported and discussed herein are supported by studies with robust methodological designs, clearly defined diagnostic criteria, and well-described outcomes. Studies classified as low quality were exclusively case reports, which inherently present important methodological limitations due to their design. However, their inclusion does not significantly impact the overall high-quality profile of the evidence base. The predominance of methodologically sound and well-reported studies enhances confidence in the conclusions drawn and strengthens the reliability of the interpretations presented. Therefore, it can be inferred that the available evidence consistently highlights patterns of biological relevance and clinical significance in the context of the feline HCM phenotype. Although most studies demonstrated high methodological quality according to the JBI tools, a subset of studies (*n* = 18/63) reported manufacturer-related funding, editorial roles, or potential conflicts of interest associated with the evaluated biomarkers. Although 18 of the included studies reported manufacturer-related funding or potential conflicts of interest, the overall conclusions of this review were based on the body of evidence as a whole and were not derived exclusively from these studies. Similar findings regarding the diagnostic and prognostic utility of NT-proBNP and cTnI were also reported in studies without declared conflicts of interest, supporting the robustness of the conclusions presented. Nevertheless, these potential conflicts of interest should be taken into consideration when interpreting the available evidence. These factors may represent potential sources of bias and should be considered when interpreting the reported diagnostic and prognostic performance of the biomarkers. Industry involvement may influence study design, assay selection, cutoff determination, data interpretation, and the reporting of biomarker performance. This may indirectly favor the perceived performance of specific biomarkers. Therefore, the available evidence should be interpreted with caution, particularly when comparing the diagnostic and prognostic accuracy of commercially available assays across different studies.

Among the analyzed studies, a higher frequency of affected cats was observed in middle-aged to older individuals. According to previously reported evidence on the HCM phenotype, the mean age at diagnosis varies depending on the characteristics of the studied population; however, it most commonly affects cats older than 6 years (Payne et al. 2015; Fox et al. 2018, 2019; Novo Matos et al. 2022; Seo et al. 2025; Li et al. 2025). Furthermore, age ≥ 6 years has been identified as a predictor of feline HCM (Payne et al. 2010; Novo Matos and Payne 2023). A substantial proportion of studies reported the breeds of affected cats, including Maine Coon, Ragdoll, Persian, Domestic Shorthair, and mixed-breed cats, all of which have been previously described as predisposed to HCM, particularly the Maine Coon and Ragdoll breeds (Meurs et al. 2005, 2007; Payne et al. 2010, 2015; Fox et al. 2018, 2019; Ontiveros et al. 2019; Novo Matos et al. 2022, 2023a; Seo et al. 2024b, 2025). Some studies also reported the genotypic profile of affected cats, frequently identifying homozygous mutations (Borgeat et al. 2015c; Langhorn et al. 2016); homozygous individuals may exhibit more severe disease expression (Ontiveros et al. 2019).

Regarding the demographic profile, a higher prevalence of affected male cats, both intact and neutered, was observed. Consistent with previous findings in feline HCM, males appear to be more frequently affected, although this observation remains incompletely understood (Freeman et al. 2015; Payne et al. 2015; Pierce et al. 2017; Harris et al. 2017; Langhorn et al. 2018; Ward et al. 2018; Novo Matos et al. 2023a; Seo et al. 2025). A possible explanation involves the presence of X-linked genes within the genetic profile, such as lysosome-associated membrane protein 2 (LAMP2) and alpha-galactosidase (GLA), which could theoretically contribute to greater phenotypic expression in males (Raffle et al. 2025). However, this observation should be interpreted with caution, as sex-related differences may also reflect sampling bias, differences in study inclusion, or potential hormonal influences.

Among the studies involving observational evaluation of animals, excluding those focused on molecular or genetic investigations, the presence of clinical signs was commonly reported. According to Luis Fuentes et al. (2020) and de Sousa et al. (2025) cats with HCM predominantly exhibit cardiovascular manifestations. Clinical signs of CHF and/or ATE were frequently observed in affected animals (Liu et al. 2020; Ironside et al. 2021; Li et al. 2023; Chong et al. 2024). However, it is important to note that some studies also included subclinical cats (van Hoek et al. 2020a; Hoek et al. 2020b; Coleman et al. 2020; Kaplan et al. 2023; Chong et al. 2024). Asymptomatic animals represent a significant diagnostic challenge due to the absence of clinical signs indicative of HCM, which may hinder early detection, monitoring strategies, and therapeutic decision-making (Luis Fuentes and Wilkie 2017; Gaia de Sousa et al. 2025). The presence of CHF and/or ATE is generally associated with disease progression and reflects underlying hemodynamic decompensation. Frequently, ATE is often associated with activation of Virchow’s triad, which may lead to partial or complete vascular obstruction (Hogan et al. 2015; Payne et al. 2015; Hogan 2017; Bakirel et al. 2021; Liu et al. 2021; Johnson et al. 2024; Brainard et al. 2025; Oranges et al. 2026).

Of the 63 studies analyzed, only ten (Coleman et al. 2020; Bakirel et al. 2021; Kaya and Bakırel 2022; Fries et al. 2022; Stack et al. 2023; Chong et al. 2024; Neumann 2025; Carter et al. 2026; Satomi et al. 2026; Lidbury et al. 2026) classified affected cats according to the staging system proposed by the ACVIM in A-D stage (Luis Fuentes et al. 2020). Classification of animals according to disease stage allows for a more accurate assessment of disease progression, incorporating both clinical and laboratory findings. In this context, evaluating biomarker use according to disease stage could help determine the magnitude of biomarker elevation and its potential implications for clinical progression. In many of the studies reviewed, however, staging was not explicitly reported, and cats were broadly categorized as either subclinical (B1 or B2) or clinical (C or D), representing an important limitation. Furthermore, it should be noted that even within the ACVIM framework, differentiation between stages B1 and B2 may be challenging due to the reliance on terms such as “low” and “high” risk for CHF and/or ATE, despite recommendations to consider additional parameters (Luis Fuentes et al. 2020).

It is important to consider that the clinical classifications of the animals obtained in the included studies were heterogeneous and not consistently aligned with the ACVIM staging system. Most studies did not apply a standardized ACVIM A-D classification, which may be partly related to the fact that this framework was formally consolidated in 2020. Instead, broader categories, such as subclinical or preclinical disease, compensated or decompensated cardiomyopathy, and congestive heart failure were used. Although these categories show some overlap with the ACVIM stages, they do not allow for a direct or systematic stratification between the studies. While it is possible to pre-classify the groups based on biomarker concentrations (e.g., higher levels of NT-proBNP and cTnI in CHF and advanced forms), a formal and reliable analysis of the animals based on the ACVIM consensus was not feasible. This represents a methodological limitation related to heterogeneity in the classification and reporting of the HCM phenotype across studies. Although the lack of adherence to the ACVIM staging system represented a methodological limitation among the included studies, it is important to recognize that the ACVIM classification was primarily developed to guide clinical management of HCM. Cats assigned to the same ACVIM stage may still present relevant differences in disease expression, particularly regarding the degree of myocardial injury, compensatory mechanisms, and disease progression. These aspects may not be fully captured by echocardiographic staging alone. In this context, circulating biomarkers (e.g., NT-proBNP and cTnI) may provide additional information that complements the current staging system, allowing a more refined stratification of affected cats. Furthermore, they may help identify differences in severity and prognosis among cats within the same ACVIM stage. This may be particularly relevant in borderline situations such as ACVIM stages B1 and B2, where biomarker concentrations, interpreted alongside clinical and echocardiographic findings, may support more informed clinical decision-making.

The analysis of cardiovascular biomarkers constituted the main focus of this review, particularly NT-proBNP and cardiac troponin I (cTnI), which have been previously described as available laboratory options for the feline HCM phenotype. According to Ahmad et al. (2023), biomarkers can be measured to assess the presence and magnitude of pathophysiological processes (e.g., normal or altered), as well as in response to the monitoring of therapeutic strategies. It is important to emphasize that biomarkers may be used in clinical practice in both quantitative and/or qualitative formats. In situations where only qualitative testing is available, it is important to verify the cut-off values, since the test provides only categorical results (positive or negative). Particularly in Brazil, de Sousa et al. (2025) highlighted that their use in clinical practice is limited by several factors, with cost being the main one. Therefore, in Brazil, echocardiography is more commonly used, as it is more effective and economically viable (de Sousa et al. 2025).

The most commonly used biomarkers in clinical routine for diagnostic support in cats with HCM are NT-proBNP and cTnI, being present in almost 75% of the studies identified in the systematic search. Of all the studies evaluated, 28 reported NT-proBNP as a diagnostic aid for the HCM phenotype. NT-proBNP is considered a biomarker of myocardial function and is predominantly released by ventricular cardiomyocytes in response to increased myocardial wall stress and chamber stretch (Borgeat et al. 2015b; Gavazza et al. 2021; de Sousa et al. 2025). Its release is triggered by conditions such as pressure overload, diastolic dysfunction, and elevated ventricular filling pressures, all of which are commonly observed in cats with HCM (Borgeat et al. 2015c; Luis Fuentes et al. 2020; Gavazza et al. 2021; de Sousa et al. 2025). Early studies involving NT-proBNP in cats with the HCM phenotype showed satisfactory results for severe forms, including in those carrying mutations in the MYBPC3 gene, but failed to detect mild or moderate cases (Maclean et al. 2006; Hsu et al. 2009; Singh et al. 2010). However, in later years, NT-proBNP began to be studied in milder forms of the disease. Changes in cut-off values to > 100 pmol/L were associated with high diagnostic performance for mild HCM, supporting its clinical utility (Wess et al. 2011). A considerable number of studies did not report cut-off values for NT-proBNP, which may compromise its precision and clinical applicability.

Other benefits of NT-proBNP have been described, such as differentiation between occult disease and primary/secondary cardiomyopathies, suggesting that it may have diagnostic value when used appropriately and at the correct time (Fox et al. 2011; Abdelhaleem et al. 2025). It is important to emphasize that interpreting biomarker cutoff values requires caution and application within the clinical context. Although reducing biomarker cutoff thresholds may improve test sensitivity, modifications in specificity may occur, leading to an increased frequency of false-positive results and compromising overall test performance. Furthermore, in screening situations such as routine evaluations, pre-anesthetic assessments, or in cats without evident signs of cardiovascular abnormalities, animals may be referred for more detailed evaluations prematurely, which may influence healthcare costs and contribute to overestimating the presence of the phenotype. Therefore, the diagnostic performance of a biomarker should be considered according to the intended purpose of its use. Careful clinical interpretation is required to avoid overdiagnosis and over-referral in low-risk cats, where biomarker elevations may not reflect clinically significant disease.

To date, NT-proBNP is described as one of the most commonly used biomarkers (Hezzell et al. 2016; Pierce et al. 2017; Ward et al. 2018), although it may be influenced by extracardiac conditions and is not recommended for use in healthy animals due to its high variability (Harris et al. 2017; Wurtinger et al. 2017). In healthy cats, the use of NT-proBNP and positive test results should be interpreted with caution. Cats with positive NT-proBNP results do not necessarily present structural disease or relevant conditions. In addition, they may not demonstrate significant changes from a clinical point of view, which may not represent an evident problem regarding anesthetic risk, clinical management, or long-term prognosis. However, such findings may lead to additional diagnostic investigations, which influences the financial costs involved and the owner’s emotional well-being, without a defined clinical benefit. In screening scenarios, especially during pre-anesthetic evaluations, a positive biomarker result in cats with mild or moderate disease may imply the need for additional complementary examinations, without necessarily representing a relevant increase in the anesthetic risk to which the patient will be subjected. In these situations, performing examinations such as echodopplercardiography may not significantly influence anesthetic planning or clinical outcome. Thus, diagnostic costs may be increased, in addition to generating more emotional impact on owners. Overall, the available evidence supports NT-proBNP as a useful adjunctive biomarker across different stages of the HCM phenotype, provided that results are interpreted together with clinical and echocardiographic findings (de Sousa et al. 2025).

Based on the evidence described for NT-proBNP, this biomarker generally presents favorable utility in predefined clinical conditions. The use of NT-proBNP in animals presenting with dyspnea may be valuable in differentiating the origin of respiratory distress (cardiac or non-cardiac). A study conducted by Ward et al. (2018) demonstrated that the use of this biomarker presents satisfactory diagnostic performance for CHF in patients with respiratory difficulty. In addition, the combination of POC NT-proBNP and VETBlue demonstrated moderate sensitivity and high specificity for the diagnosis of CHF in cats with respiratory distress, reinforcing its clinical utility in emergency triage settings (Ward et al. 2018). Another important application is the evaluation of cats presenting with heart murmurs, considered one of the most frequent indications for biomarker use, followed by arterial thromboembolism and anesthetic screening (O’Shaughnessy et al. 2021). Furthermore, NT-proBNP may contribute to risk stratification and monitoring of cats with HCM or suspected cases, especially in the presence of structural cardiac abnormalities or CHF. Overall, although NT-proBNP has limitations, it may be clinically useful depending on the intended objective and clinical context.

The reviewed studies evaluating NT-proBNP demonstrated a consistent increase in its concentrations in cats with CHF, as well as the ability to differentiate healthy animals from those with structural heart disease, including ACVIM stages B and C (Liu et al. 2021; Lu et al. 2021; Stack et al. 2023). Correlations with other biomarkers were also observed, both positive and negative, suggesting its involvement in different pathophysiological pathways (Hanås et al. 2020; Liu et al. 2021; Abdelhaleem et al. 2025). NT-proBNP does not appear to be significantly influenced by birth weight, body condition score, age range, or breed (Hanås et al. 2020), but it is associated with echocardiographic variables indicative of structural remodeling, such as IVSd, LVFWd, and LAE (Hanås et al. 2020; Seo et al. 2020). The presence of SAM is also described as a relevant factor to be considered when interpreting biomarker results (Seo et al. 2020).

The studies also reported that elevated NT-proBNP values are associated with greater disease severity and poorer prognosis. An increased risk of CHF, ATE, and sudden death may be associated with higher NT-proBNP concentrations (Ironside et al. 2021; Stack et al. 2023). Values > 1500 pmol/L may indicate poorer prognosis, while decreasing or stable values are associated with better survival outcomes (Liu et al. 2021). More recently, Abdelhaleem et al. (2025) showed that NT-proBNP may also vary in cases of primary (greater increase) and secondary cardiomyopathies, reflecting fluctuations regardless of etiology. Although not included in the present systematic search, the recent study by Carter et al. (2026), showed that the association of population profile (breed and sex), clinical examination, and NT-proBNP in asymptomatic cats with heart murmurs may be useful in identifying the presence of cardiomyopathies. Therefore, NT-proBNP remains a valid biomarker in cats with HCM, assisting in diagnosis, evaluation of the etiology of dyspnea, and prognosis, particularly in cases with structural cardiac abnormalities.

The cTnI was another biomarker widely reported in 32 studies, recognized as a marker of myocardial injury, with increased serum levels associated with processes involving structural cardiac damage, including atrial remodeling and injury caused by regurgitant jets. Regarding cTnI, this biomarker is a structural protein of the cardiac contractile apparatus and is widely recognized as a biomarker of myocardial injury (Gavazza et al. 2021). In feline HCM, its release into the circulation is associated with cardiomyocyte injury secondary to increased myocardial wall stress, microvascular ischemia, myocardial fibrosis, and adverse cardiac remodeling (Langhorn et al. 2014; Hori et al. 2018; Luis Fuentes et al. 2020; Ferasin et al. 2020; Gavazza et al. 2021; Lidbury et al. 2026). Since 2002, studies have demonstrated the diagnostic benefits of cTnI in the HCM phenotype, with elevated concentrations observed in affected patients (Herndon et al. 2002). The cTnI concentrations show a considerable increase in patients with moderate to severe disease, due, for example, to conditions such as fibrosis and remodeling (Herndon et al. 2002). However, it should be noted that values may also be influenced in healthy animals (e.g., breed and sex) (Hanås et al. 2022) and by other conditions such as hyperthyroidism (Sangster et al. 2014; Janus et al. 2023).

Evidence indicates that cTnI may be associated with clinical outcomes and can be considered a prognostic marker (Langhorn et al. 2014; Borgeat et al. 2015b), particularly due to its higher levels in cardiac patients, especially those with CHF (cut-off 0.163 ng/mL) (Hori et al. 2018). Higher cut-off values (> 0.234 ng/mL) appear to improve diagnostic performance, although with reduced specificity (Hori et al. 2018). Other studies have shown that cTnI values tend to be altered in cases of SAM, especially in the presence of LVOTO and pressure overload (Hertzsch et al. 2019; van Hoek et al. 2020b; Satomi et al. 2025). It has also been reported that cTnI concentrations are associated with echocardiographic variables reflecting the magnitude of hemodynamic impairment, such as LVFWTd and the LA/Ao ratio (Hanås et al. 2022). The agreement between cTnI and echocardiographic variables was also observed in several studies included in this systematic review, particularly with LA/Ao, LVOTO, and LAE (van Hoek et al. 2020a; Seo et al. 2020; Bakirel et al. 2021; Hanås et al. 2022; Satomi et al. 2025).

An interesting aspect observed in some articles included in this systematic search was the ability of cTnI concentrations to decrease following the implementation of dietary or therapeutic strategies, which may be associated with a reduction in myocardial injury (van Hoek et al. 2020b; Saponaro et al. 2025). The study by van Hoek et al. (2020b) demonstrated reductions in cTnI concentrations after one year of a diet enriched with protein and supplemented with fatty acid sources (eicosapentaenoic acid—EPA and docosahexaenoic acid—DHA). Furthermore, Saponaro et al. (2025) highlighted that cTnI values may be modifiable after the initiation of therapeutic management for acute myocardial injury in cats with HCM and ATE. Although not included in the present systematic search, evidence suggests that cats with transient myocardial thickening, such as that associated with stress-induced hypertrophy, may also show increased cTnI concentrations (Novo Matos et al. 2018).

In pre-anesthetic screening scenarios, the use of cTnI may represent a potential adjunctive option for the assessment of myocardial injury. However, evidence supporting its use as a standalone diagnostic tool for anesthetic risk stratification remains limited. The evidence gathered in this review indicates that varying degrees of elevation can be observed in cats with significant structural heart disease or other conditions (e.g., hyperthyroidism), whether compensated or decompensated (Langhorn et al. 2014, 2019; Hertzsch et al. 2019; Ferasin et al. 2020; van Hoek et al. 2020b; Hanås et al. 2022; Satomi et al. 2025; Lidbury et al. 2026). Therefore, the specificity of cTnI in pre-anesthetic screening contexts is limited. Accordingly, cTnI should not be used in isolation to guide the selection of anesthetic protocols or justify the postponement of procedures in asymptomatic patients. Nevertheless, it is important to note that elevated cTnI concentrations may raise concern, and although they do not imply a disproportionate increase in perceived clinical risk, they may warrant more thorough cardiovascular investigation. It should also be emphasized that the combination of biomarkers with imaging techniques increases diagnostic accuracy (Carter et al. 2026). Overall, cTnI is better characterized as a marker of myocardial injury and disease severity rather than a primary tool for predicting anesthetic risk (Langhorn et al. 2014, 2019; Hori et al. 2018; Ferasin et al. 2020; Lidbury et al. 2026).

In this context, the studies included in this systematic review demonstrated that cTnI concentrations increase progressively according to disease severity, supporting its use in risk stratification (Langhorn et al. 2016, 2019; Hori et al. 2018; Liu et al. 2020; Ferasin et al. 2020; Hanås et al. 2022; Stack et al. 2023). In addition, the observed correlation between cTnI and NT-proBNP suggests that myocardial cellular injury occurs concomitantly with increased wall stress (Liu et al. 2020; Abdelhaleem et al. 2025). Higher cTnI concentrations were observed in obstructive phenotypes and in the presence of SAM and LVOTO, with a direct association with the magnitude of obstruction, reinforcing the role of mechanical stress in biomarker release (Seo et al. 2020; Satomi et al. 2025). Furthermore, the association of cTnI with echocardiographic variables, such as LVFWd and LA/Ao, supports its relationship with structural remodeling and atrial overload, as previously discussed (Seo et al. 2020; Hanås et al. 2022). There may also be an interaction between myocardial injury and systemic inflammation, as evidenced by positive correlations between cTnI and acute-phase proteins, expanding the interpretation of this biomarker beyond a purely mechanical componente (Liu et al. 2020). Finally, the evidence suggests that cTnI may assist in differentiating between primary and secondary cardiomyopathies, highlighting its potential as a complementary diagnostic tool (Abdelhaleem et al. 2025).

An important limitation of the studies is the absence of serial measurements of biomarker concentrations, particularly NT-proBNP and cTnI. Only two studies reported serial data for NT-proBNP and cTnI; however, the measurement intervals were 6 and 12 months (van Hoek et al. 2020b; Kaplan et al. 2023). It was frequently observed that biomarkers were generally used during the initial evaluation to characterize the overall clinical status, whereas animals were reassessed without further biomarker measurements being performed. The scarcity of studies reporting serial biomarker concentrations in the context of HCM is considered concerning, as it limits the understanding of the phenotype dynamics. The lack of serial monitoring hinders a better understanding of phenotypic progression, differentiation between acute and chronic conditions, therapeutic evaluation and monitoring, as well as prognostic and conclusive inferences. Considering only single measurements represents an important methodological limitation, as they may not reliably reflect the current clinical scenario. Therefore, isolated measurements should be interpreted with caution, as they may reflect transient variations or hemodynamic adjustments. In this context, serial measurements could provide more comprehensive information regarding disease magnitude, pathological progression, or structural remodeling.

In general, in addition to classical biomarkers of myocardial injury and stress, several serum, metabolic, protein, and molecular markers have been investigated in cats with HCM and were identified in 26 studies. These biomarkers reflect distinct biological pathways involved in HCM progression, including extracellular matrix remodeling, inflammation, endothelial dysfunction, metabolic regulation, and gene expression (Fonfara et al. 2021; Joshua et al. 2023; Guelfi et al. 2024; Chong et al. 2024). Studies have reported associations between markers of myocardial injury and systemic biochemical alterations. From a molecular perspective, preliminary evidence suggests changes in gene expression and microRNAs, involving pathways related to hypoxia, inflammation, tissue remodeling, and cardiac hypertrophy. Inflammatory mediators, growth factors, and proteins associated with the extracellular matrix and protein metabolism regulation have also been described, some of which are increased in cats with HCM compared to healthy animals. Together, these findings suggest that feline HCM involves complex and multifactorial biological mechanisms, and that these biomarkers, although still in early stages of investigation, contribute to expanding the understanding of the molecular, inflammatory, and structural processes involved.

Although biomarkers provide relevant data for clinical practice, particularly in feline cardiology and the HCM phenotype, echocardiography should still be considered the gold standard for diagnostic confirmation. The need for echocardiographic evaluation may be indicated by point-of-care (POC) testing results. Positive NT-proBNP POC tests often suggest myocardial involvement and indicate the need for further investigation for cardiomyopathy (Ward et al. 2018; Loughran et al. 2019; Hanås et al. 2020; Lu et al. 2021). The combination of biomarkers with imaging tools such as radiography and echocardiography may be valuable during the diagnostic process. In the study by Laudhittirut et al. (2020), NT-proBNP POC testing was the best isolated method; however, its accuracy increased progressively when combined with radiographic parameters, such as the product of cardiac length and width. The authors also observed that the most effective method was the measurement of the left atrium by echocardiography, especially when combined with NT-proBNP. The association of Vertebral Heart Score, echocardiography, and NT-proBNP achieved an accuracy close to 97%, demonstrating that combining diagnostic tools substantially increases reliability (Laudhittirut et al. 2020).

The variability in cut-off values, absence of serial measurements, physiological variations, and factors such as sensitivity, specificity, and positive/negative predictive values indicate that biomarkers still require further clarification. Several studies included in this systematic review demonstrated that biomarkers supporting their integration into multimodal cardiovascular assessment (Laudhittirut et al. 2020; Hanås et al. 2020, 2022; Liu et al. 2020; Seo et al. 2020; van Hoek et al. 2020b; Kaya and Bakırel 2022; Li et al. 2023; Satomi et al. 2025).

From a practical standpoint, the use of cardiovascular biomarkers may be considered a valuable option in routine clinical practice, provided that their limitations, intended applications, and contextual interpretation are carefully considered. In general, biomarker testing in healthy patients (low risk) is not strongly recommended due to the increased likelihood of false-positive results, which may lead to unnecessary referrals to specialists and increased financial costs. In intermediate-risk populations, such as cats with a heart murmur or those undergoing pre-anesthetic evaluation, lower cut-off values may be useful to increase sensitivity and support rule-out strategies, although this occurs at the expense of reduced specificity. In contrast, in high-risk or symptomatic cats (e.g., dyspnea, CHF, or HCM), higher cut-off values and serial assessments may improve specificity and support risk stratification and prognostic evaluation, while contributing to more accurate clinical decision-making. Biomarkers may provide guidance in clinical practice, however, inherent limitations related to their performance and interpretation must be considered.

Although not included in the present systematic review due to the adopted temporal cutoff, studies published in 2026 reinforce the relevance of cardiac biomarkers in feline medicine. It was observed that the association between breed, sex, clinical evaluation, and NT-proBNP concentrations in asymptomatic cats presenting heart murmurs increased the accuracy of heart disease detection, with an approximate sensitivity of 87% and specificity of 61%. These findings suggest potential usefulness in the screening of patients referred for echocardiography, reducing unnecessary costs and owner apprehension (Carter et al. 2026). Furthermore, elevated NT-proBNP concentrations were associated with the presence of arterial thromboembolism (ATE) in cats with obstructive hypertrophic cardiomyopathy, with a proposed cut-off value of 491 pmol/L, showing 96% sensitivity and 93.5% specificity (Carter et al. 2026). Values above this threshold were associated with atrial enlargement, more pronounced ventricular hypertrophy, lower FS%, and a greater presence of spontaneous echocardiographic contrast, demonstrating potential prognostic value in identifying patients at greater risk for ATE (Oranges et al. 2026).

A novel assay for cTnI measurement in healthy and cardiopathic cats was also validated, demonstrating significantly higher concentrations in animals with HCM compared to healthy cats. These findings reinforce the clinical applicability of cTnI as an auxiliary biomarker in differentiating healthy from diseased animals (Lidbury et al. 2026). Additionally, high doses of carvedilol in cats with ACVIM B1 obstructive HCM promoted improvement in the left ventricular outflow tract, myocardial function, and reduction of cTnI concentrations, suggesting therapeutic benefit in these patients (Satomi et al. 2026). Overall, these recent findings further support the clinical and prognostic relevance of cardiac biomarkers in HCM phenotype.

In general, biomarkers are widely used as minimally invasive tools that may reflect different aspects of cardiac function and disease processes, including hemodynamic status, myocardial injury, and prognosis (Netala et al. 2025). In human cardiology, they are commonly applied in clinical contexts requiring rapid assessment, such as acute coronary syndromes and myocardial infarction, often in combination with other diagnostic modalities (Netala et al. 2025). In veterinary cardiology, biomarkers are investigated across different cardiac conditions, including feline cardiomyopathies, as part of multimodal diagnostic approaches.

## Conclusion

This systematic review summarizes the current evidence regarding cardiovascular biomarkers in cats with the HCM phenotype. Overall, NT-proBNP and cardiac troponin I were the most frequently studied biomarkers and showed consistent associations with different aspects of disease expression. In screening and clinical triage settings, particularly in cats with respiratory signs or heart murmurs, biomarkers were mainly used to support the differentiation between cardiac and non-cardiac disease and to identify patients requiring further cardiac evaluation. With respect to structural cardiac disease, biomarker concentrations were generally associated with echocardiographic markers of hypertrophy, LA enlargement, and disease severity, although they did not replace imaging for phenotypic confirmation. From a prognostic perspective, higher biomarker concentrations were commonly associated with more advanced disease, CHF, and adverse outcomes, suggesting a potential role in risk stratification. However, important limitations remain, including heterogeneity across studies, variability in cut-off values, and limited longitudinal assessment. Future research should focus on standardized protocols, stage-based analyses, and serial biomarker evaluation to better define their clinical utility in feline HCM.

## Supplementary Information

Supplementary Material 1.

## Data Availability

The datasets generated during and/or analysed during the current study are available from the corresponding author on reasonable request. The data extraction table for the articles included in the analysis is available in the Online Resource 1.
