# Supplementary material for: Cardiovascular biomarkers in feline hypertrophic cardiomyopathy phenotype: evidence from the last decade
Source: Vet Res Commun. 2026 Jul 17;50(5):465. doi: 10.1007/s11259-026-11408-9 (PMC13379500; doi:10.1007/s11259-026-11408-9)
Supplement: Supplementary file 1 — Supplementary Material 1. [file 11259_2026_11408_MOESM1_ESM.docx]

| Supplementary Table 1. Characteristics and data extraction from studies included in the systematic review (2015-2026) | | | | | | | | | | | | | | | | | | | | | | | | | | | | | |
| --- | --- | --- | --- | --- | --- | --- | --- | --- | --- | --- | --- | --- | --- | --- | --- | --- | --- | --- | --- | --- | --- | --- | --- | --- | --- | --- | --- | --- | --- |
| Year  Study Design | **Animals** | **HCM** | **Biomarkers** | | | | | | | | | | | | | | | | | | | | | | | | | **Findings** | **Reference** |
| 2015  Research | - 48 felines, 2 groups  - Ragdolls  (G1) – genotype + (n=24); 7.12 (2.17–12.5) years; 10 (42%) M  (G2) – genotype – (n=24); 7.61 (1.00–14.3) years; 6 (25%) M | - | NTproBNP (pmol/L) | | | | | | | | | | | | G1 – 43.5 (30.5–105) | | | | | | | | | | | | | - The evaluation of CITP and PIINP using human assay kits after validation in feline serum demonstrated satisfactory performance. No detection of CITP was observed in any sample.  - PIINP showed correlation with LA/Ao and a trend toward association with LAD; however, no correlation was observed with CITP use.  - CITP concentrations were associated with genotype and NT-proBNP in univariate analysis. In multivariable analysis, only genotype remained significant.  - The study demonstrated differences in CITP levels in cats carrying MYBPC3 mutations, even in the absence of ventricular hypertrophy. Furthermore, it highlights the occurrence of alterations in collagen turnover, especially type I collagen remodeling. | Borgeat et al. (2015)  DOI: 10.1111/jsap.12332 |
|  |  |  |  |  |  |  |  |  |  |  |  |  |  |  | G2 – 38.5 (27.0–65.5) | | | | | | | | | | | | |  |  |
|  |  |  | cTnI (ng/mL) | | | | | | | | | | | | G1 – 0.01 (0.01–0.02) | | | | | | | | | | | | |  |  |
|  |  |  |  |  |  |  |  |  |  |  |  |  |  |  | G2 – 0.01 (0.01–0.01) | | | | | | | | | | | | |  |  |
|  |  |  | CITP | | | | | | | | | | | | Values not available | | | | | | | | | | | | |  |  |
|  |  |  | PIIINP | | | | | | | | | | | | Values not available | | | | | | | | | | | | |  |  |
| 2015  Research | - 64 felines, 2 groups  - All cats (n=64); 5.5 (1.0-14.3) years; 36% M  (G2) – MYBPC3 + (n=24); 6.16 (1.0-12.9) years; 46% M  (G3) – MYBPC3 – (n=40); 5.25 (1.0-14.3) years; 20% M | Some HCM ACVIM (stage not described) | NTproBNP (pmol/L) | | | | | | | | | | | | (G1) 39.5 (24-1500) | | | | | | | | | | | | | - cTnI concentrations were higher in cats positive for the MYBPC3 mutation, whereas no difference was observed for NT-proBNP concentrations.  - No differences in biomarker concentrations were identified among cats carrying ADRB1 and ACE polymorphisms. | Borgeat et al. (2015)  DOI: 10.1016/j.jvc.2015.06.005 |
|  |  |  |  |  |  |  |  |  |  |  |  |  |  |  | (G2) 43.5 (24-1500) | | | | | | | | | | | | |  |  |
|  |  |  |  |  |  |  |  |  |  |  |  |  |  |  | (G3) 36.5 (24-125) | | | | | | | | | | | | |  |  |
|  |  |  | cTnI (ng/mL) | | | | | | | | | | | | (G1) 0.01 (0.01-0.46) | | | | | | | | | | | | |  |  |
|  |  |  |  |  |  |  |  |  |  |  |  |  |  |  | (G2) 0.01 (0.01-0.46) | | | | | | | | | | | | |  |  |
|  |  |  |  |  |  |  |  |  |  |  |  |  |  |  | (G3) 0.01 (0.01-0.03) | | | | | | | | | | | | |  |  |
|  |  |  | ACE and ADRB1 polymorphism genotyping | | | | | | | | | | | | | | | | | | | | | | | | |  |  |
| 2015  Research | - 28 cats, 2 groups  - All cats (n=28); 5.2 ± 1.1 years; 20 NM and 8 NF.  (G1) – LVH + (n=14)  (G2) – LVH – (n=14)  - Breeds: Domestic short- and  Longhairs (n = 11), Birman (n = 5), Somali (n = 4), Maine coon (n = 4), Siamese (n = 2), Exotic shorthair (n=1) and Sphinx (n=1). | Some HCM ACVIM (stage not described) | NT-proBNP (pmol/L)  < 100 | | | | | | | | | | | | (All cats) 40 (<24 to 162) | | | | | | | | | | | | | - In multivariable analysis, NT-proBNP and IGF-1 were associated with left ventricular hypertrophy.  - The study demonstrated that an increase of 50 units in NT-proBNP resulted in a 0.04-unit increase in IVSd, whereas an increase of 500 units in IGF-1 resulted in a 0.10-unit increase in IVSd.  - Measurement of IGF-1 in growing cats is recommended to better evaluate its influence on HCM. | Freeman et al. (2015)*  DOI: 10.1111/jvim.12503 |
|  |  |  |  |  |  |  |  |  |  |  |  |  |  |  | (G1) 44 (<24 to 162) | | | | | | | | | | | | |  |  |
|  |  |  |  |  |  |  |  |  |  |  |  |  |  |  | (G2) 26 (<24 to 105) | | | | | | | | | | | | |  |  |
|  |  |  | Insulin-like growth factor-1 (lg/L)  <350 | | | | | | | | | | | | (All cats) 482 (181–838) | | | | | | | | | | | | |  |  |
|  |  |  |  |  |  |  |  |  |  |  |  |  |  |  | (G1) 536 (329–838) | | | | | | | | | | | | |  |  |
|  |  |  |  |  |  |  |  |  |  |  |  |  |  |  | (G2) 412 (181–689) | | | | | | | | | | | | |  |  |
|  |  |  | Glucose (mg/dL)  60–110 | | | | | | | | | | | | (All cats) 85 (70–100) | | | | | | | | | | | | |  |  |
|  |  |  |  |  |  |  |  |  |  |  |  |  |  |  | (G1) 80 (70–90) | | | | | | | | | | | | |  |  |
|  |  |  |  |  |  |  |  |  |  |  |  |  |  |  | (G2) 90 (80–100) | | | | | | | | | | | | |  |  |
|  |  |  | Insulin (µU/L)  10–40 | | | | | | | | | | | | (All cats) 7 (3–22) | | | | | | | | | | | | |  |  |
|  |  |  |  |  |  |  |  |  |  |  |  |  |  |  | (G1) 8 (4–16) | | | | | | | | | | | | |  |  |
|  |  |  |  |  |  |  |  |  |  |  |  |  |  |  | (G2) 6 (3–22) | | | | | | | | | | | | |  |  |
| 2015  Research | - 31 felines, 2 groups  (G1) Healthy cats (n=18); 3 (1-10) years; 8 NM/10 NF  (G2) Cardiomyopathy (n=13); 9 (1-15) years; 10 NM/3 NF  - All cats - domestic longhair or shorthair breeds. | Some HCM ACVIM (HOCM or HCM) | NT-proBNP (pmol/L) | | | | | | | | | | | | (G1) ≤1.8 pmol/L | | | | | | | | | | | | | - The median proBNP concentration in cats with symptomatic cardiomyopathy is higher than in asymptomatic cats.  - The findings highlight that the C-terminal fraction of proBNP can be detected in feline plasma at low picomolar concentrations, which are lower than those described for the N-terminal fraction. | Solter et al. (2015)  DOI: 10.1016/j.tvjl.2015.07.015 |
|  |  |  |  |  |  |  |  |  |  |  |  |  |  |  | (G2) 15.6 pmol/L (1.7 - 78.8)  Asymptomatic cardiomyopathy [11.4 pmol/L (1.7–36.8)]  Symptomatic cardiomyopathy  [24.6 pmol/L (10.1–78.2)] | | | | | | | | | | | | |  |  |
| 2016  Case Reports | - 96 felines, 2 groups  (G1) HCM cats (n=61)  25 Maine Coon [5 (6 months-10) years; 3F/5NF/2M/15NM; 13 homozygote, 2 heterozygote]  29 British shorthair [4 (1-14) years; 3F/1NF/2M/23NM]  7 Norwegian forest cats [5 (1-8) years; 2F/1M/4 NM]  (G2) Healthy cats (n=35)  8 Maine Coon [2.5 (11 months - 6) years; 5F/1NF/2M]  21 British shorthairs [3(1-10) years; 13F/2NF/5M/1NM]  6 Norwegian forest [8.5 (4-12) years; 2F/4NF] | HCM ACVIM (stage not described) | cTnI (values not described) | | | | | | | | | | | | | | | | | | | | | | | | | - In G1, British Shorthair cats showed higher cTnI values than Maine Coons.  - Regarding genotype pattern, homozygous Maine Coons presented higher values than heterozygous cats.  - No differences were identified within G2. | Langhorn et al. (2016)  DOI: 10.1136/vr.103549 |
| 2016  research | - 43 felines, 3 groups, 4-8.1 kg  (G1) Healthy (n=21); 11-50 months  (G2) Unequivocal (n=18); 25-92 months  (G3) LVH (n=4); 21-79 months | Some HCM ACVIM (stage not described) | proANP (nmol/l) | | | | | | | | | | | | (G1) 0.13 ± 0.23 | | | | | | | | | | | | | - proANP is not recommended for early stages of HCM.  - There was variability in proANP concentrations among cats in G1.  - There were no significant differences between cats in G1 and G2.  - proANP levels in G2 increased as body weight increased (r = 0.304). This was also observed in G3 (r = 0.5578).  - proANP levels in G3 were higher than in G1 and G2.  - In G3, there was a correlation with ejection fraction and left ventricular systolic diameter.  - In none of the groups was proANP correlated with left ventricular wall thickness. | Parzeniecka-Jaworska et al. (2016)  DOI: 10.1515/pjvs-2016-0101 |
|  |  |  |  |  |  |  |  |  |  |  |  |  |  |  | (G2) 0.09 ± 0.18 | | | | | | | | | | | | |  |  |
|  |  |  |  |  |  |  |  |  |  |  |  |  |  |  | (G3) 0.28 ± 0.60 | | | | | | | | | | | | |  |  |
| 2017  research | - 349 felines,  - Heart disease (n=80); 11 (1–22) years  - Control (n=269); 12 (2–24) years  - Subgroups (CHF + or -)  (G1) CHF +, alive 30d  (G2) CHF +, dead 30d  (G3) CHF -, alive 30d  (G4) CHF -, dead 30d  (G5) CHF +, alive 60m  (G6) CHF +, dead 60m  (G7) CHF -, alive 60m  (G8) CHF -, dead 60m | Some HCM ACVIM (stage not described) | RDW (all cats) | | | | | | | | | | | | (G1) 16.5 (15.7–17.6) | | | | | | | | | | | | | - Single RDW values were not predictive of mortality but may help identify cats with decompensated heart disease and CHF.  - Cats with CHF had higher RDW values than cats without CHF (16.3% vs 15.8%; P = 0.02).  - No significant association was found between RDW tertiles and 30-day or 6-month mortality.  - RDW was significantly higher in cats with UCM (16.3% vs 15.8%; P = 0.03). | Roderick et al. (2017)  DOI: 10.1177/1098612X16649988 |
|  |  |  |  |  |  |  |  |  |  |  |  |  |  |  | (G2) 15.7 (15.2–17.0) | | | | | | | | | | | | |  |  |
|  |  |  |  |  |  |  |  |  |  |  |  |  |  |  | (G3) - | | | | | | | | | | | | |  |  |
|  |  |  |  |  |  |  |  |  |  |  |  |  |  |  | (G4) 15.7 (14.8–16.8) | | | | | | | | | | | | |  |  |
|  |  |  |  |  |  |  |  |  |  |  |  |  |  |  | (G5) 16.4 (15.6–17.6) | | | | | | | | | | | | |  |  |
|  |  |  |  |  |  |  |  |  |  |  |  |  |  |  | (G6) 16.1 (15.3–17.1) | | | | | | | | | | | | |  |  |
|  |  |  |  |  |  |  |  |  |  |  |  |  |  |  | (G7) 15.8 (15.1–16.7) | | | | | | | | | | | | |  |  |
|  |  |  |  |  |  |  |  |  |  |  |  |  |  |  | (G8) 15.7 (15.0–16.9) | | | | | | | | | | | | |  |  |
|  |  |  | HCM cats | | | | | | | | | | | | 15.80 (14.30–22.50); n = 75 | | | | | | | | | | | | |  |  |
| 2017  Research | - 53 felines, 2 groups, 6 (1–17) years, 3F/16NF/32NM  (G1) Heart disease (n=26)  (G2) Control (n=27)  - Various breeds: Domestic shorthairs, Sphynx, Maine Coon,  Bambino, American Bobtail, Bengal, Devon Rex, Persian, and Siamese. | Some HCM ACVIM (stage not described) | NT-proBNP (pmol/L) | | | | | | | | | | | | (G1) 561.5 (32–2,077) | | | | | | | | | | | | | - The median NT-proBNP concentration in G1 was higher than in G2.  - An abnormal POC ELISA result was associated with a median NT-proBNP concentration of 854 pmol/L (204–2,077). A normal POC ELISA result was associated with a median NT-proBNP concentration of 29 pmol/L (4–173).  - The POC ELISA showed 65.4% sensitivity and 100% specificity, with a PPV of 100% and NPV of 75%.  - A cutoff value of 100 pmol/L showed 84.6% sensitivity and 100% specificity, with a PPV of 100% and NPV of 87.1% for differentiating diseased cats from controls.  - NT-proBNP concentrations in cats with CHF ranged from 82 to 171 pmol/L. | Harris et al. (2017)*  DOI: 10.1111/jvim.14776 |
|  |  |  |  |  |  |  |  |  |  |  |  |  |  |  | (G2) 23 (4–94) | | | | | | | | | | | | |  |  |
|  |  |  |  |  |  |  |  |  |  |  |  |  |  |  | POC ELISA –  36 normal/17 abnormal | | | | | | | | | | | | |  |  |
| 2017  Research | - 25 cardiac samples, 18 HCM and 7 control  - Majority cats: DSH | HCM ACVIM (stage not described) | Myofibrillar protein expression, troponin T and I, and Ca^2+^ sensitivity. | | | | | | | | | | | | | | | | | | | | | | | | | - Myofibrillar protein expression was normal, except for MyBP-C haploinsufficiency.  - Cats with HCM showed increased Ca²⁺ sensitivity (2.06 ± 0.13-fold), and was not affected by troponin I phosphorylation.  - Replacement with wild-type troponin T or addition of EGCG restored modulation of Ca²⁺ sensitivity. | Messer et al. (2017)  DOI: 10.3389/fphys.2017.00348 |
| 2017  Research | - 31 felines, 3 times of evaluation  - 9.0 ± 4.3 years, 24NM/7NF.  - Various breeds: Domestic short- and longhaired cats (n = 27), Cornish rex, Maine Coon cat, Oriental shorthair and Siamese (n=1, each).  (G1) Admission  (G2) Discharge  (G3) Re-evaluation | Majority HCM ACVIM (stage not described) | NT-proBNP (pmol/L) | | | | | | | | | | | | (G1) 1,713 (160 – 3,784) | | | | | | | | | | | | | - There was a significant reduction in NT-proBNP across time points, particularly between admission–discharge and admission–re-evaluation.  - Only pimobendan was associated with a decrease in NT-proBNP, with significantly lower concentrations observed in cats that received the drug compared to those that did not.  - Survival time was longer in cats that showed greater reductions in NT-proBNP. | Pierce et al. (2017)  DOI: 10.1111/jvim.14690 |
|  |  |  |  |  |  |  |  |  |  |  |  |  |  |  | (G2) 902 (147–3,223) | | | | | | | | | | | | |  |  |
|  |  |  |  |  |  |  |  |  |  |  |  |  |  |  | (G3) 1,124 (111–2,727) | | | | | | | | | | | | |  |  |
| 2018  Research | - 161 felines, 2 groups  (G1) Control (n=78); 0.5-18.0 years, 38F/40M  (G2) Cardiomyopathy (n=83); 0.3-22.0 years; 53M/30F  - Various breeds – majority DSH | Some HCM ACVIM (stage not described) | Plasma ANP (pg/ml) | | | | | | | | | | | | (G1) 43.3 (IQR 33.0- 56.3) | | | | | | | | | | | | | - ANP values increase as the disease progresses.  - ANP concentrations differed between control cats and cats with cardiomyopathy (LAD+, LAD−, or HF).  - Values >77.5 pg/mL showed 66.3% sensitivity and 84.6% specificity for differentiating control cats from cats with cardiomyopathy.  - A cutoff of 110.9 pg/mL demonstrated 73.6% sensitivity and 93.5% specificity for differentiating cats with cardiomyopathy (LAD+/HF) from LAD− cats.  - Differentiation of HF from all cats showed 85.2% sensitivity and 85.1% specificity, using a cutoff of 118.6 pg/mL.  - ANP >58.5 pg/mL showed 71.2% sensitivity and 76.9% specificity for differentiating healthy cats from those with cardiomyopathy (excluding restrictive and unclassified forms). | Heishima et al. (2018)  DOI: 10.1016/j.jvc.2018.04.008 |
|  |  |  |  |  |  |  |  |  |  |  |  |  |  |  | (G2/LAD+) 144.7 (58.0 264.6) | | | | | | | | | | | | |  |  |
|  |  |  |  |  |  |  |  |  |  |  |  |  |  |  | (G2/LAD-) 65.8 (42.2-92.7) | | | | | | | | | | | | |  |  |
|  |  |  |  |  |  |  |  |  |  |  |  |  |  |  | (G2/HF) 197.7 (143.9-471.4) | | | | | | | | | | | | |  |  |
| 2018  Research | - 181 felines, 2 groups  (G1) Control (n=88), 0.3–16.0 years, 43M/45F  (G2) HCM (n=93), 0.5–19.0 years, 73M/20F  - Various breeds – majority DSH | HCM ACVIM (stage not described) | cTnI (ng/mL) | | | | | | | | | | | | (G1) 0.027 (IQR, 0.012-0.048) | | | | | | | | | | | | | - cTnI concentrations differ between healthy and diseased cats and have been described as a supportive diagnostic marker.  - cTnI concentrations were higher in diseased cats.  - Cats with HF also showed higher concentrations compared with asymptomatic cats.  - Variables related to wall/septal thickness and thyroxine levels were predictors of troponin concentrations.  - The distinction between asymptomatic/severely affected cats and controls showed 62% sensitivity and 100% specificity, using a cutoff of 0.163 ng/mL.  - Differentiation between cats with and without LAD, using a cutoff of 0.213 ng/mL, showed 84.6% sensitivity and 84.9% specificity.  - A cutoff of 0.234 ng/mL showed 95% sensitivity and 77.8% specificity for differentiating cats with and without HF. | Hori et al. (2018)  DOI: 10.1111/jvim.15131 |
|  |  |  |  |  |  |  |  |  |  |  |  |  |  |  | (G2 asymptomatic) 0.103 [IQR, 0.042-0.345] | | | | | | | | | | | | |  |  |
|  |  |  |  |  |  |  |  |  |  |  |  |  |  |  | (G2 LAD) 0.305 [IQR, 0.182-0.500] | | | | | | | | | | | | |  |  |
|  |  |  |  |  |  |  |  |  |  |  |  |  |  |  | (G2 HF) 1.703 [IQR, 0.376-4.383] | | | | | | | | | | | | |  |  |
| 2018  Research | - 52 felines, 3 groups  (G1) Renal (n=19), 11 (2–17) years, 1F/7NF/11NM  (G2) Cardiac (n=19); 5 (1–11) years, 1F/4NF/14NM  (G3) Control (n=14); 7.5 (3–16) years, 5F/5NF/1M/3NM  - Various breeds. | HCM ACVIM (stage not described) | cTnI (ng/mL) | | | | | | | | | | | | (G1) 0.052 (0.015–0.78) | | | | | | | | | | | | | - cTnI concentrations were higher in the G1 and G2 groups compared with controls, with no differences observed between the diseased groups.  - Primary cardiac diseases are not the only conditions associated with increased cTnI concentrations. | Langhorn et al. (2018)  DOI: 10.1177/1098612X18813427 |
|  |  |  |  |  |  |  |  |  |  |  |  |  |  |  | (G2) 0.083 (0.003–3.27) | | | | | | | | | | | | |  |  |
|  |  |  |  |  |  |  |  |  |  |  |  |  |  |  | (G3) 0.012 (0.003–0.14) | | | | | | | | | | | | |  |  |
| 2018  Research | - 94 felines, 4 groups  (G1) CKD (n=17), 11 (4–17) years, 7NF/10NM  (G2) HCM (n=40), 6.5 (1–15) years, 4F/9NF/4M/23NM  (G3) DM (n=17), 9 (6–16) years, 5NF/1M/11N  (G4) Control (n=20), 7 (3–15) years, 3F/11NF/6NM  - Various breeds | Some HCM ACVIM cats (stage not described) | SDMA (µg/dL) | | | | | | | | | | | | (G1) 19 (10–93) | | | | | | | | | | | | | - SDMA concentrations in G1 were higher than in the other groups.  - No differences were observed between G2 and G4.  - G3 showed the lowest SDMA values.  - HCM did not influence SDMA concentrations. | Langhorn et al. (2018)  DOI: 10.1111/jvim.14902 |
|  |  |  |  |  |  |  |  |  |  |  |  |  |  |  | (G2) 9 (4–24) | | | | | | | | | | | | |  |  |
|  |  |  |  |  |  |  |  |  |  |  |  |  |  |  | (G3) 7 (3–11) | | | | | | | | | | | | |  |  |
|  |  |  |  |  |  |  |  |  |  |  |  |  |  |  | (G4) 10 (5–15) | | | | | | | | | | | | |  |  |
| 2018  Research | - 51 felines, 2 groups with respiratory distress  (G1) CHF (n=33), 10.5 ± 3.6 years, 25/33 (75.8) M  (G2) No cardiac (n=18), 10.0 ± 4.1 years, 11/18 (61.1) M  - Various breeds – majority of DSH | Some HCM ACVIM (stage not described) | Blood NT-proBNP positive, n (%) | | | | | | | | | | | | (G1) 31/33 (93.9) | | | | | | | | | | | | | - The combination of >1 site evaluated using the VETBlue+ protocol and blood NT-proBNP showed 75.8% sensitivity and 88.9% specificity for diagnosing CHF (multivariate analysis).  - Use of the NT-proBNP SNAP test may generate false-positive results.  - NT-proBNP showed 93.9% sensitivity and 72.2% specificity for diagnosing CHF in patients with respiratory distress (univariate analysis).  - Blood samples are preferred over pleural fluid samples due to the moderate-to-high occurrence of false-positive results. | Ward et al. (2018)*  DOI: 10.1111/jvim.15246 |
|  |  |  |  |  |  |  |  |  |  |  |  |  |  |  | (G2) 5/18 (27.8) | | | | | | | | | | | | |  |  |
|  |  |  | Pleural effusion NT-proBNP positive, n (%) | | | | | | | | | | | | (G1) 11/12 (91.7) | | | | | | | | | | | | |  |  |
|  |  |  |  |  |  |  |  |  |  |  |  |  |  |  | (G2) 3/7 (42.9) | | | | | | | | | | | | |  |  |
| 2019  Research | - 41 felines, 3 groups  (G1) Compensated HCM (n=15), 6 (1–14.5) years, 4F/11M  (G2) Decompensated HCM (n=16), 9.5 (1.5–16.5) years, 4F/12M  (G3) Control (n=10), 4.25 (1–7.5) years, 5F/5M  - Various breeds – majority European Shorthair | HCM ACVIM (stage not described) | POC cTnI | | | | | | | | | | | | (G1) 0.29 (0.0–1.16) | | | | | | | | | | | | | - Cats with decompensated HCM showed higher cTnI concentrations.  - Cats that died from cardiogenic causes within one year had mean cTnI concentrations of 0.27 ng/mL compared with cats with HCM that remained alive after one year. | Bartoszuk et al. (2019)  DOI: 10.1016/j.tvjl.2018.11.005 |
|  |  |  |  |  |  |  |  |  |  |  |  |  |  |  | (G2) 0.05 (0.0–0.38) | | | | | | | | | | | | |  |  |
|  |  |  |  |  |  |  |  |  |  |  |  |  |  |  | (G3) All 0.00 | | | | | | | | | | | | |  |  |
| 2019  Research | - 289 felines, 3 groups  (G1) Control (n=148), 7 (4-10) years, 68F/80M  (G2) Heart disease (n=102), 10 (7-12) years, 29F/73M  (G3) Equivocal (n=39), 10 (7-14) years, 17F/22M  - Various breeds – majority DSH. | Some HCM ACVIM (stage not described) | POC-BNP (values/results not described) | | | | | | | | | | | | | | | | | | | | | | | | | - POC-BNP associated with clinical evaluation showed moderate agreement with the cardiologist’s diagnosis.  - The inclusion of POC-BNP did not result in a significant improvement in diagnostic agreement compared with the previous assessment stage.  - After performing the POC-BNP test, 62.7% of the cats were correctly diagnosed by the non-specialist clinician.  - The diagnostic performance observed after POC-BNP was similar to that previously obtained with FCU, with no relevant improvement in diagnostic accuracy. | Loughran et al. (2019)  DOI: 10.1111/jvim.15549 |
| 2019  Research | - 166 felines, 6 groups  (G1) Control (n=87), 2.75 (1.5–5.92) years, 34M/53F  (G2) Equivocal (n=15), 3.33 (1.58-8.58) years, 11M/4F  (G3) Mild HCM (n=16),9.92 (1.56–12.92) years, 11M/5F  (G4) Moderate HCM (n=10),11.46 (3.31–12.58) years, 8M/2F  (G5) Severe HCM (n=34), 6.38 (4.46–12.56) years, 32M/2F  (G6) ATE (n=4), 5.46 (2.15-6.52) years,1M/3F  - Various breeds | HCM ACVIM (stage not described) | cTnI (ng/mL) | | | | | | | | | | | | (G1) 0.013 (0.006-0.025) | | | | | | | | | | | | | - cTnI concentrations - lower in the healthy groups (G1 and G2) compared with the cardiomyopathy groups.  - G5 showed higher values than G3, and G6 demonstrated the highest cTnI concentrations. Among cats in G5, decompensated animals had higher concentrations than compensated cats.  - A positive correlation - cTnI concentrations, LVFWT, and the LA/Ao ratio.  - A cutoff value >0.06 ng/mL differentiated healthy cats from cats with cardiomyopathy with 91.7% sensitivity and 95.4% specificity, and also identified severe asymptomatic forms with 100% sensitivity.  - cTnI - excellent diagnostic performance. | Hertzsch et al. (2019)  DOI: 10.1111/jvim.15498 |
|  |  |  |  |  |  |  |  |  |  |  |  |  |  |  | (G2) 0.022 (0.013-0.037) | | | | | | | | | | | | |  |  |
|  |  |  |  |  |  |  |  |  |  |  |  |  |  |  | (G3) 0.1 (0.028-0.58) | | | | | | | | | | | | |  |  |
|  |  |  |  |  |  |  |  |  |  |  |  |  |  |  | (G4) 0.174 (0.121-0.356) | | | | | | | | | | | | |  |  |
|  |  |  |  |  |  |  |  |  |  |  |  |  |  |  | (G5) 0.760 (0.407-2.53) | | | | | | | | | | | | |  |  |
|  |  |  |  |  |  |  |  |  |  |  |  |  |  |  | (G6) 6.413 (4.336-11.266) | | | | | | | | | | | | |  |  |
| 2020  Research | - 59 felines, 2 groups  (G1) Healthy (n=27); 5 (2.5-8.0) years  (G2) Subclinical HCM (n=32); 6.0 (3.5 - 7.0) years  -(G1) 16M/27F; (G2) 24M/8F  -Various breeds, with more mixed  -G2 subdivided in treatment and placebo | (G2) B2 |  | | *Treatment* | | | | | | | | | | | | | *Placebo* | | | | | | | | | | - No significant changes in biomarkers before and after atenolol.  - There was no evidence of a reduction in the concentration of circulating biomarkers with the use of atenolol. | Coleman et al. (2020)*  DOI: 10.1016/j.jvc.2020.06.002 |
|  |  |  | *cTnI* | | D0: <0.2 | | | | | | | | | | | | | D0: <0.2 | | | | | | | | | |  |  |
|  |  |  |  |  | D180: <0.2 | | | | | | | | | | | | | D180: <0.2 | | | | | | | | | |  |  |
|  |  |  | *NT-proBNP* | | D0: 229 | | | | | | | | | | | | | D0: 161 | | | | | | | | | |  |  |
|  |  |  |  |  | D180: 235 | | | | | | | | | | | | | D180: 147 | | | | | | | | | |  |  |
| 2020  Research | - 58 felines – data and radiography, 2 groups  (G1) Healthy (n=20); 10M/10F  (G2) Cardiac diseases (n=38); 20M/18F  -Various breeds  -Different types of diagnostic options performed | HCM in the cardiac disease group (majority) | NT-proBNP alone or combined with other methods such as radiography and ultrasonography | | | | | | | | | | | | | | | | | | | | | | | | | - Best diagnostic option: Point-of-care NT-proBNP.  - Greater accuracy was found when combining point-of-care NT-proBNP with the product of cardiac length × width via radiography.  - Even higher accuracy was obtained when point-of-care NT-proBNP was combined with cardiac length × width plus left atrial size measured either by radiography or echocardiography.  - The best method was left atrial echocardiography in cats with HCM. Accuracy improved further when combined with NT-proBNP.  - VHS + echocardiography + NT-proBNP reached nearly 97% accuracy.  - The more tests are combined, the higher the diagnostic reliability, although echocardiography is still required for definitive confirmation. | Laudhittirut et al. (2020)  DOI:  10.14202/vetworld.2020.872-878 |
| 2020  Research | - 57 felines, 3 groups  (G1) CHF (n=25); 8.1 ± 4.7 (1.0-15.3) years; 8F/17M  (G2) Preclinical (n=12); 6.6 ± 4.4 (0.7-14.3) years; 3F/9M  (G3) Healthy (n=20); 4.3 ± 3.0 (0.6-11.3) years; 10F/10M | HCM in the cardiac disease group (majority) | NT-proBNP | | | | | | | | | (G1) 780 (578–1477)  (G2) >1500 (1308–>1500) | | | | | | | | | | | | | | | | - Cats with CHF had higher concentrations of both cardiac and renal biomarkers. NT-proBNP was elevated in CHF cats compared with preclinical cardiomyopathy. NT-proBNP differed between healthy and preclinical cats.  - LRG1, SAA, and ceruloplasmin were higher in CHF versus healthy cats. SAA and ceruloplasmin were higher in CHF versus preclinical cats.  - Moderate correlation between NT-proBNP, SDMA and creatinine. Strong correlation between NT-proBNP and cTnI. Acute phase proteins (LRG1, SAA, ceruloplasmin, AGP) showed positive correlations with NT-proBNP, cTnI, atrial size, and CHF class. AGP had a strong correlation with cTnI and was a predictor of poor prognosis in CHF. Moderate correlations were observed for LRG1, SAA, ceruloplasmin with LA/Ao ratio, atrial diameter, or both. SAA and ceruloplasmin showed moderate correlations with CHF stage.  - Approximately 70% of CHF cats had NT-proBNP >1500 pmol/L. All cats that died had NT-proBNP >1500 pmol/L. Among survivors, 80% had stable NT-proBNP (increase <60% or reduced). | Liu et al. (2020)*  DOI: 10.1111/jvim.15757 |
| 2020  Research | - 1 Feline Maine Coon without A31P mutation  - Evaluated in serial exams (10 to 24 months) | HCM | Novel homozygous intronic variant in TNNT2 | | | | | | | | | | | | | | | | | | | | | | | | | - Discovery of a novel homozygous intronic variant in TNNT2 associated with HCM and early-onset CHF. - The TNNT2 gene may be considered a potential genetic biomarker for HCM in Maine Coon cats. | McNamara et al. (2020)*  DOI: 10.3389/fphys.2020.608473 |
| 2020  Research | -139 felines, 3 groups  (G1) Healthy (n=100); 4.6 (2.1-8.7) years; 57F/43M  (G2) HCM without LAE (n=32); 5.3 (3.6-9.1) years; 12F/20M  (G3) HCM with LAE (n=7); 6.6 (3.0-9.0) years; 1F/6M  - (G1): 35NF/22F/33NM/10M  - (G2): 9NF/3F/16NM/4M  - (G3): 0NF/1F/6NM/0M  -(G1): Norwegian Forest, Birman, and Domestic Shorthair cats.  -(G2): Various breeds, including Maine Coon, Persian, Ragdoll, among others. | HCM ACVIM  (stage not described) | *Biomarker* | | | | | | | *G1* | | | | | | | | *G2* | | | | | | | | *G3* | | - Cutoff ≥100 pmol/L:  - Detecting control compared with HCM: ELISA, visual inspection, and automated POCT evaluation showed specificity of 72% and 74%; sensitivity of 97%, 98%, and 95%, respectively.  - Detecting HCM: ELISA and visual inspection and automated POCT evaluation showed specificity of 100% and sensitivity of 97%, 98%, and 95%, respectively.  - Detecting HCM with and without LAE: specificity of 69% and sensitivity of 97%, 98%, and 95%.  -NT-proBNP levels were higher in males.  -Age, birth weight, body condition score, or breed did not affect NT-proBNP levels in healthy cats.  -Increased IVSd and LVFWd increased NT-proBNP levels.  -POCt and ELISA showed similar sensitivity and specificity.  -HCM + LAE cats showed ELISA and POCt abnormalities.  -Systolic blood pressure values ​​were lower in animals with higher NT-proBNP.  -Associations with elevated NT-proBNP were found in cats with hypertrophy, LAE, or combinations. | Hanås et al. (2020)  DOI: 10.1111/jvim.15754 |
|  |  |  | *NT-proBNP pmol/L(ELISA)* | | | | | | NF <24(<24-39)  B <24(<24-38)  DSH <24(<24-29) | | | | | | | | | 253 (IQR 52-456) pmol/L | | | | | | | | 1496 (1007 ->1500) pmol/L | |  |  |
|  |  |  | *POC test Normal/abnormal visual evaluation* | | | | | | 98/2 | | | | | | | | | 10/22 | | | | | | | | 0/7 | |  |  |
|  |  |  | *POC test Normal/abnormal automated evaluation* | | | | | | 95/5 | | | | | | | | | 29/10 | | | | | | | | | |  |  |
| 2020  Case Report | - Feline, 14-year-old  - Neutered male  - Domestic shorthair  - CHF signs and electric alterations | HCM end-stage | *cTnI* | | | | | | | 0.41 ng/mL (reference < 0.15 ng/mL) | | | | | | | | | | | | | | | | | | -The findings revealed a condition of acute myocardial injury.  -Therapeutic management was employed, but biomarker measurements were not performed serially. | Sarcinella et al. (2020)  DOI 10.2460/javma.256.9.984 |
| 2020  Research | - 47 felines, 3 groups  (G1) Healthy (n=15); 6.43(2.87-12.25) years; 13F:2M  (G2) Occult HCM (n=17); 6.68(1.67-12.65) years; 6F:11M  (G3) HCM + CHF (n=15); 7.37(2.0-15.5) years; 3F:12M | HCM ACVIM (stage not described) | NT-proBNP | | | | | | | | | | | | | | | | | | | | | | | | | - NT-proBNP measurements were performed using a second-generation ELISA, with 1500 pmol/L being the upper limit of detection. | Rohrbaugh et al. (2020)  DOI: 10.1111/jvim.15777 |
|  |  |  | N/A | | | | | | 496 (24-1500)  (n=16) | | | | | | | | | 1500(496-1500)  (n=13) | | | | | | | | | |  |  |
| 2020  Case Report | -Feline, 10-month-old,  - Neutered male  - Norwegian Forest  -Carvedilol-disopyramide  -Carvedilol: Start and gradually increase to 0.30 mg/kg twice daily.  -Disopyramide: 5.4 mg/kg orally twice daily, then increase to 10.9 mg/kg orally twice daily. | HCM with obstruction | *NT-proBNP* | | | | | | | | | | | | | | | | | | | | | | | | | -There was a reduction in NT-proBNP concentrations following co-therapy with carvedilol and disopyramide. At the final assessment (D190), concentrations remained approximately five times above the reference value.  - The authors hypothesized that the decrease in the left ventricular outflow tract pressure gradient may have contributed to the observed reduction in NT-proBNP levels. | Hori et al. (2020)  DOI: 10.1016/j.jvc.2020.04.002 |
|  |  |  | D1: > 1500 | | | | | | | | | | | | | | | | | | | | | | | | |  |  |
|  |  |  | D19:499 | | | | | | | | | | | | | | | | | | | | | | | | |  |  |
| 2020  Case Report | - Feline, 7-year-old, spayed female and Domestic longhair.  - ATE signs. | HCM + thrombus | cTnI 0.71 (reference 0-0.11 ng/mL)  blood coagulation profile normal | | | | | | | | | | | | | | | | | | | | | | | | | - cTnI not investigated in serial measurements.  - aPTT target of 1.5–2 times baseline.  - No values reported. | Vezzosi et al. (2020)  DOI: 10.1016/j.jvc.2020.03.002 |
| 2020  Research | -44 felines, 2 groups  (G1) Test (n=23); 4.3 (1.42-17) years; 17M/6F  (G2) Control (n=21); 7.9 (2-13.9) Years; 14M/7F  - Various breeds  - Serial evaluations (baseline, 6 and 12 months | Subclinical HCM |  | | | *Test group (G1)* | | | | | | | | | | | | *Control group (G2)* | | | | | | | | | | - Cats show reductions in cTnI and IGF-1 after one year of using the test diet (starch restriction, increased protein, and EPA + DHA supplementation, in addition to changes in free nitrogen extract levels). - IGF-1 decreased along with the reduction in hypertrophy. - The diet may modify cTnI and IGF-1 concentrations, being a potential influencer of myocardial thickness and cardiomyocyte hypertrophy. | van Hoek et al. (2020)  DOI: 10.1111/jvim.15925 |
|  |  |  | NT-proBNP | | | Baseline: 211 (24-1500)  6 months: 98 (24-1500)  12 months: 150 (26-1500) | | | | | | | | | | | | | Baseline: 271 (26-1500)  6 months: 199.5 (24-1500)  12 months: 263 (24-1500) | | | | | | | | |  |  |
|  |  |  | cTnI | | | Baseline: 0.16 (0.02-9.49)  6 months: 0.07 (0.01-0.55)  12 months: 0.12 (0.01-.56) | | | | | | | | | | | | | Baseline: 0.14 (0.01-1.74)  6 months: 0.1 (0.01-2.25)  12 months: 0.1 (0.01-2.35) | | | | | | | | |  |  |
|  |  |  | IGF-1 | | | Baseline: 460 (216-772)  6 months: 369 (118-658)  12 months: 401 (73.3-720) | | | | | | | | | | | | | Baseline: 448 (207-786)  6 months: 463 (66.5-956)  12 months: 498 (132-1000) | | | | | | | | |  |  |
| 2020  Research | -51 felines, median age 4.42 years.  -27 cats had atrial dilation; median age 4.65 years.  - 11 had systemic hypertrophy; median age 6.1 years.  - 7 presented both; median age 7.5 years.  - Various breeds. | Asymptomatic HCM | All group | | | | | | | | | | Insulin: 22 (3.9–113 µU/mL)  Glucose: 5.9 (3.8–11.2 mmol/L)  IGF-1: 405 (81.2–786 ng/mL)  NT-proBNP: 179 (26–1489 pmol/L)  cTnI: 0.17 (0.01–16.45 ng/mL)  SAA: 0 (0–21.8 mg/L) | | | | | | | | | | | | | | | - No associations were observed between insulin and IGF-1 with echocardiographic parameters and cardiac biomarkers – to be investigated. - Body weight and body condition score of cats with HCM are associated with insulin, IGF-1, and glucose concentrations. -NT-proBNP and cTnI are associated with Doppler echocardiographic parameters. | van Hoek et al. (2020)*  DOI: 10.1111/jvim.15730 |
|  |  |  | LA remodelling | | | | | | | | | | Insulin: 21.5 (6.3–76 µU/mL) Glucose: 5.9 (3.8–9.5 mmol/L) IGF-1: 461 (81.2–786 ng/mL) NT-proBNP: 350 (27–1489 pmol/L) cTnI: 0.32 (0.02–16.5 ng/mL) SAA: 0 (0–21.8 mg/L) | | | | | | | | | | | | | | |  |  |
|  |  |  | Generalized hypertrophy | | | | | | | | | | Insulin: 16.7 (6–53 µU/mL) Glucose: 5.9 (4.2–9.5 mmol/L) IGF-1: 482 (81.2–730 ng/mL) NT-proBNP: 290 (42–1489 pmol/L) cTnI: 0.33 (0.02–16.5 ng/mL) SAA: 0.1 (0–21.8 mg/L) | | | | | | | | | | | | | | |  |  |
| 2020  Research | - 140 felines, 2 groups  (G1) HCM + SAM (n=70); 4.1 [2.4-7.0] years; 45M (42 NM) / 25F (22NF)  (G2) HCM – SAM (n=70); 6.9 [4.0-10.6] years; 49M (42 NM)/ 21F (18 NF)  - Various breeds | HCM |  | | | | | | *G1* | | | | | | | | | *G2* | | | | | | | | | | - G1 showed higher concentrations of NT-proBNP and cTnI, observed in cats with and without DLVOTO.  - For NT-proBNP and cTnI, the explanatory variables were SAM, CHF, maximum wall thickness, and atrial dimension.  - The authors suggest that the presence of SAM should be considered when interpreting feline biomarkers in HCM. | Seo et al. (2020)*  DOI: 10.1111/jvim.15807 |
|  |  |  | NT-proBNP (pmoL/L) | | | | | | 729 [275–1467]  (n = 61) | | | | | | | | | 65 [25–271]  (n = 58) | | | | | | | | | |  |  |
|  |  |  | cTnI (ng/mL) | | | | | | 0.27 [0.10–0.81]  (n = 59) | | | | | | | | | 0.07 [0.01–0.43]  (n = 64) | | | | | | | | | |  |  |
|  |  |  | POC cTnI | | | | | | 7/59 (11.9%) | | | | | | | | | 7/64 (10.9%) | | | | | | | | | |  |  |
| 2021  Research | - 67 felines, 2 groups  (G1) HCM (n= 17 cats; 5 with atrial thrombus); 7.3 (± 4.3) years  (G2) 50 cats without cardiac diseases (10 control, 6 lymphoma, 34 systemic inflammatory state - 18 FIP); 10 (± 6.1) years  -(G1) Mostly neutered males  -(G2) Mostly neutered females | HCM ACVIM (stage not described) | HCM compared with other diseases | | | | | | | | | | | | | | | | | | | | | | | | | - There is variation in inflammatory and remodeling markers among diseases.  - The sex of the feline may influence cardiovascular response and outcome.  - Active inflammation is not related to structural remodeling in HCM particularly MMP-2 MMP-3 and TIMP-3.  - Myocardial changes may occur through regional activation of TIMP-1 and TIMP-2. | Fonfara et al. (2021)  DOI: 10.1016/j.rvsc.2021.03.027 |
|  |  |  | *IL-1** | | | | | | | | | | | 0.81 (0.46–1.43) | | | | | | | | | | | | | |  |  |
|  |  |  | *IL-6** | | | | | | | | | | | 0.39 (0.14–1.08) | | | | | | | | | | | | | |  |  |
|  |  |  | *IL-18** | | | | | | | | | | | 54.38 (25.36–116.61) | | | | | | | | | | | | | |  |  |
|  |  |  | *TGF-β** | | | | | | | | | | | 58.36 (39.47–86.27) | | | | | | | | | | | | | |  |  |
|  |  |  | *MMP-2** | | | | | | | | | | | F 13.37 (5.1–35.0)  M 142.83 (83.5–240.9) | | | | | | | | | | | | | |  |  |
|  |  |  | *MMP-3** | | | | | | | | | | | F 1.37 (0.60–3.13)  M 6.73 (4.55–9.96) | | | | | | | | | | | | | |  |  |
|  |  |  | *MMP-9** | | | | | | | | | | | 0.20 (0.10–0.40) | | | | | | | | | | | | | |  |  |
|  |  |  | *MMP-13** | | | | | | | | | | | 0.194 (0.027–1.42) | | | | | | | | | | | | | |  |  |
|  |  |  | *TIMP-1** | | | | | | | | | | | 0.048 (0.014–0.162) | | | | | | | | | | | | | |  |  |
|  |  |  | *TIMP-2** | | | | | | | | | | | 952.24 (575.96–1499.2) | | | | | | | | | | | | | |  |  |
|  |  |  | *TIMP-3** | | | | | | | | | | | F 3.77 (0.83–17.14)  M 1159.2 (442.53-3036.46) | | | | | | | | | | | | | |  |  |
|  |  |  | HCM (a) vs HCM + AT (b) | | | | | | | | | | | | | | | | | | | | | | | | |  |  |
|  |  |  |  | | *Atria* | | | | | | | | | | | *Ventricle* | | | | | | | | | | | |  |  |
|  |  |  | *IL-6** | | a) 2.14 (0.57–3.71)  b) 32.6 (0.01–80.01) | | | | | | | | | | | | a) 1.02 (0.23–1.81)  b) 10.85 (0.05–22.17) | | | | | | | | | | |  |  |
|  |  |  | *IL-8** | | a) 12.53 (0.82–24.25)  b) 120.32 (0.05–246.6) | | | | | | | | | | | |  | | | | | | | | | | |  |  |
|  |  |  | *TGF-β** | | a) 228.5 (109.8–347.2)  b) 621.1 (95.5–1512.2) | | | | | | | | | | | |  | | | | | | | | | | |  |  |
|  |  |  | *TIMP-3** | | a) 6248 (3569–8927)  b) 7727 (1661–13,793) | | | | | | | | | | | |  | | | | | | | | | | |  |  |
| 2021  Research | - 257 felines  - Median age: 10.4 years (0.1-21.7).  -Various breeds.  -Data by electronic health record | - | *NT-proBNP* | | | | | | | | | | | | | | | | | | | | | | | | | - Heart murmur was the most common reason for NT-proBNP testing, followed by thromboembolism and weight loss. - NT-proBNP was rarely used to differentiate between cardiac and non-cardiac causes. - It is used for screening cats for occult cardiomyopathies and suspected arterial thromboembolism (ATE).  -Serial measurements: recommendation  -Lack of knowledge among veterinarians. - Diagnostic accuracy and utility may be affected, and further studies are needed. | O’Shaughnessy et al. (2021)*  DOI: 10.1002/vetr.945 |
| 2021  Research | - 33 felines, 2 groups  (G1) HCM (n=9); 6.9 years  (G2) HCM + ATE* (n=24); 5.5 years  - 70% male  - No breed definition  - ATE episode within 12h | (G1) B1  (G2) C | *cTnI ** | | | | | | | | | (G1) 0.559±0.3229  (G2) 13.860±64.203 | | | | | | | | | | | | | | | | (G1) Alive; (G2) 15/23 died  -All animals: Positive correlation: CK-MB with LVd, cTnI and LA/AO, LA and CK_MB, LA and NTproBNP, cTnI and CK-MB. Negative correlation: LVOT and NT-proBNP  -In G2, positive correlation: CK_MB and IVSd, CK-MB and FS, NT-proBNP and IVSs. | Bakirel et al. (2021)  DOI: 10.9775/kvfd.2020.25073 |
|  |  |  | *cTnT* | | | | | | | | | (G1) 0.1505±0.1188  (G2) 1.695±10.657 | | | | | | | | | | | | | | | |  |  |
|  |  |  | *CK-MB** | | | | | | | | | (G1) 45.67±7.269  (G2)2490.2±1204.601 | | | | | | | | | | | | | | | |  |  |
|  |  |  | *NT-proBNP* | | | | | | | | | (G1) 33.33±10.953  (G2) 99.13±15.099 | | | | | | | | | | | | | | | |  |  |
| 2021  Research | - 217 felines after initial exclusion, 5 groups  - Median age HCM/HOCM: 9 (7-11); 20M/12F (29 neutered).  - Median age overall: 7 (4-11) | HCM/HOCM | NT-proBNP POC ELISA | | | | | | | | | | | | | | | | | | | | | | | | | - NT-proBNP POC ELISA was useful for differentiating diseased cats from healthy ones, with a sensitivity of 43%, specificity of 96%, AUC of 0.7, positive likelihood ratio of 12, negative likelihood ratio of 0.59, positive predictive value (PPV) of 78%, and negative predictive value (NPV) of 85%.  - In cats with a positive NT-proBNP ELISA, the median concentration was 303 pmol/L, which was higher compared to negative cats.  - A NT-proBNP cutoff of 99 pmol/L provided 51% sensitivity, 92% specificity, and an AUC of 0.72.  - Cutoffs of 46 pmol/L and 85 pmol/L segregated normal and abnormal cats with 74% and 59% sensitivity, and 67% and 91% specificity, respectively.  - The NT-proBNP ELISA failed in 57% of cases with distinct phenotype forms.  - A positive NT-proBNP POC is associated with heart disease, which justifies performing an echocardiographic evaluation. A negative NT-proBNP POC is not reliable for the correct exclusion of heart disease. The test is not effective as a screening tool in apparently healthy cats. | Lu et al. (2021)*  DOI: 10.1111/jvim.16156 |
| 2021  Research | - 47 felines, 3 groups  (G1) All felines (n=47); 4.0 (1.0–12.0) years  (G2) HCM with clinical CHF signs (n=15); 6.0 (1.0–12.0) years  (G3) Healthy or other signs (n=32); 4.0 (1.0–12.0) years  -Breed (pedigree/not; %):  G1 (7/40; 14.8)  G2 (2/13; 13.3)  G3 (5/27; 15.6)  -Sex (male; %): G1 (35; 74)  G2 (12; 80)  G3 (23; 71.9) | HCM | *NTproBNP (pmol/l)** | | | | | | (G1) 515 (24–1500)(n = 46)  (G2) 851 (138–1500)(n = 14)  (G3) 321 (24–1500) | | | | | | | | | | | | | | | | | | | - There was a difference in NT-proBNP concentrations between the groups, with higher values in the group that experienced an event (G2).  - When comparing reassessment values, the absolute change in NT-proBNP was smaller in G2.  - Higher NT-proBNP concentrations at the first examination of cats with preclinical HCM suggest that they may have a higher risk of CHF, ATE, or sudden death.  - NT-proBNP: AUC = 0.821 (95% CI: 0.689–0.954); Proposed cutoff value = 700 pmol/l; Sensitivity = 0.786 (95% CI: 0.524–0.924); Specificity = 0.813 (95% CI: 0.647–0.911); Positive likelihood ratio = 4.203 (95% CI: 1.938–9.063); Negative likelihood ratio = 0.263 (95% CI: 0.095–0.729).  - Although echocardiographic measurement of LA/Ao is more limited due to equipment availability, testing NT-proBNP may be an auxiliary option in cats with preclinical HCM. | Ironside et al. (2021)  DOI: 10.1177/1098612X20938651 |
|  |  |  | *cTnI (ng/ml)* | | | | | | (G1) 0.23 (0.01–23.7)(n = 46)  (G2) 0.38 (0.04–0.82)(n = 14)  (G3) 0.22 (0.01–23.7) | | | | | | | | | | | | | | | | | | |  |  |
| 2022  Research | - 96 felines, 3 groups  (G1) Healthy (n=38); 6.2 ± 1.2 years  (G2) HCM B (n=29); 6.7 ±0.75 years  (G3) HCM C (n=29); 7.5 ± 2.0 years  - No breed and sex related | HCM  B and C | *Neutrophil to lymphocyte ratio (NLR)* | | | | | | | | | | | | | | | | | | | | | | | | | - NLR: prognostic indicator for feline HCM.  - Elevated NLR reduces MST in felines with HCM Stages B and C.  - For each unit increase in NLR, the risk of death increases by 11%.  - 95% specificity for predicting death if NLR > 4.46. | Fries et al. (2022)  DOI: 10.3389/fvets.2022.813524 |
|  |  |  | G1 | | | | | | | | | G2 | | | | | | | | | | | G3 | | | | |  |  |
|  |  |  | 1.80 (0.41-8.85) | | | | | | | | | 2.48 (0.73-7.35) | | | | | | | | | | | 5.11 (1.55-48.18) | | | | |  |  |
| 2022  Research | (G1) Healthy (n=96) – Birman (n=33; 4.8 ± 4.0 years); Domestic shorthair (n=30; 7.0 ± 4.4 years) and Norwegian (n=33; 5.0 ± 3.0 years)  (G2) HCM (n=39) – 18 Domestic shorthair and other from 10 different breeds – with LAE (n=7; 6.3 ± 2.7 years); without LAE (n=32; 6.1 ± 3.4 years).  (G1) Birman (mostly females); Domestic shorthair (mostly neutered females) and Norwegian (mostly NF)  (G2) With/without LAE (mostly NM). | Preclinical HCM or clinical HCM | *Group (n)* | | | | | | | | | *cTnI* | | | | | | | | | | | *Range* | | | | | -The median serum cTnI concentration in HCM was 37 ng/L.  -cTnI was higher in cats with HCM and LAE.  -In HCM, there was a positive association of cTnI with the percentage increase in left ventricular free wall thickness during diastole and the LA/Ao ratio.  -Elevated troponin values ​​in healthy Birman cats.  -Unspayed female cats had higher values ​​when compared with intact males.  -There were differences between breed and sex.  -In HCM, cTnI increases are elevated by increased ventricular wall thickness.  -Cats with LAE had higher cTnI values ​​when compared with those without LAE. | Hanås et al. (2022)  DOI: 10.1177/1098612X221117115 |
|  |  |  | G1 (n=96) | | | | | | | | | 5.7 (2.8–11.0)* | | | | | | | | | | | <2.0–156.0 | | | | |  |  |
|  |  |  | G2 without  LAE (n=32) | | | | | | | | | 29.3 (13.3–46.5)* | | | | | | | | | | | 3.1–318.0 | | | | |  |  |
|  |  |  | G2 with LAE (n=7) | | | | | | | | | 296 (92.0–642.0)* | | | | | | | | | | | 56.0–1880.0 | | | | |  |  |
| 2022  Research | - 24 felines, 5 groups  (G1) All felines (n=24)  (G2) HCM B1 (n=7)  (G3) HCM B2 (n=6)  (G4) HCM C (n=6)  (G5) Healthy (n=5)  -10 females e 14 males | HCM B1, B2 and C | *sST2* | | | | | | | | | (G1) 24.664 ± 3.297  (G2) 24.473 ± 2.624  (G3) 30.608 ± 2.274  (G4) 25.824 ± 2.958 | | | | | | | | | | | | | | | | - There was a positive correlation between sST2 and CK-MB with left atrial diameter, between NT-proBNP and left atrial diameter, and between CK-MB, IVSd, and LVFWd with the LA/Ao ratio. - No correlation was found between sST2, NT-proBNP, Troponin I, and Troponin T. - There was no significant difference in sST2 among the groups. Further studies are needed to establish a cause–effect relationship between HCM and sST2. | Kaya and Bakırel (2022)  DOI: 10.51585/gjvr.2022.4.0044 |
|  |  |  | *NT-proBNP** | | | | | | | | | (G1) 141.43 ± 42.246  (G2) 348.00 ± 48.280  (G3) 288.17 ± 30.196  (G4) 79.20 ± 1.800 | | | | | | | | | | | | | | | |  |  |
|  |  |  | *Troponin I** | | | | | | | | | (G1) 0.06 ± 0.006  (G2) 5.03 ± 2.191  (G3) 15.68 ± 5.141  (G4) 0.04 ± 0.003 | | | | | | | | | | | | | | | |  |  |
|  |  |  | *Troponin T** | | | | | | | | | (G1) 0.027 ± 0.002  (G2) 0.151 ± 0.069  (G3) 1.777 ± 0.580  (G4) 0.016 ± 0.001 | | | | | | | | | | | | | | | |  |  |
| 2023  Research | - 1 Feline, 5-year-old, neutered male and European shorthair  - Clinical signs: weakness  - Pre-ventricular excitation | HCM ACVIM (stage not described) | cTnI: 0.22 ng/mL | | | | | | | | | | | | | | | | | | | | | | | | | - Three felines (n=17) with ventricular pre-excitation had HCM, but cTnI concentration was available in only one case. -No serial measurements were performed. - No description of cTnI variability was provided in the single case. | Sidler et al. (2023)  DOI: 10.1016/j.jvc.2023.04.005 |
| 2023  Research | -53 felines, 3 groups: in experimental phase, only 43 felines.  (G1) Placebo  (G2) Rapamycin low-dose  (G3) Rapamycin high-dose  - Of the 43 cats: NF e 37 NM, mean age was 6 years (1-12 years)  - Mostly neutered males.  -Various breeds. | Subclinical HCM | NT-proBNP | | | | | | | | | *Rapamycin group*  First visit 0.1518  D180 0.7672 | | | | | | | | | | | | | | | | - There was a positive correlation between the maximum basal wall thickness and NT-proBNP levels, with r^2^=0.26. A cutoff of 100 pmol/L was used as a reference for patients' NT-proBNP.  - Although a notable difference in maximum basal wall thickness was observed between groups stratified by baseline NT-proBNP, this difference did not reach statistical significance.  - On day 180 of rapamycin treatment, cTnI levels in the placebo group were higher compared to the treated group, but the difference was not statistically significant. | Kaplan et al. (2023)*  DOI: 10.2460/javma.23.04.0187 |
|  |  |  | cTnI | | | | | | | | | (G1) Baseline: 0.60 ng/mL (0–1.98)  D180: 491.45 pg/mL (14.63–1,996.57)  (G2) Baseline: 0.63 ng/mL (0.05–1.84)  D180: 254.39 pg/mL (9.01–808.6)  (G3) Baseline: 0.57 ng/mL (0–1.9)  D180: 244.03 pg/mL (15.02–445.9) | | | | | | | | | | | | | | | |  |  |
| 2023  Case Report | -Feline, 3-year-old  -Male neutered Ragdoll  -Clinical signs of dyspnea and tachypnea | HCM | cTnI | | | | | | | | | 2.11 (reference < 0.2-0.25) | | | | | | | | | | | | | | | | - The report described the presence of paroxysmal supraventricular tachycardia in a feline with a hypertrophic phenotype.  - Although elevated, serial measurement of troponin was not performed, with only the initial concentration at presentation being available. | Seddon et al. (2023)  DOI: 10.1177/20551169231166528 |
| 2023  Research | - 85 felines, 3 groups  (G1) Non-HCM (n=35); 6.31±4.52 years; 20M/15F  (G2) HCM (n=33); 6.43±3.63 years; 22M/11F  (G3) CATE (n=17); 7.36±3.04 years; 11M/6F | HCM | cfDNA | | | | | | | | | (G1) 8.2 ng/μL; IQR, 5.7 to 11.7  (G2) 6.6 ng/μL; IQR, 5.1 to 8.3  (G3) 11.2 ng/μL; IQR, 8.1 to 29.6 | | | | | | | | | | | | | | | | 1. cfDNA total  - G3 > G1, G2.  - G2 with CHF > G2 without CHF (11.0 ng/μL; IQR 6.4–16.8 vs 6.3 ng/μL).  2. cfDNA fragmentation  - Higher proportion <2000 bp in G3 vs G2, G1 .  - G3 > others at 36–100 bp and 101–300 bp.  3. citH3  - G2 with HCM > G2 without HCM.  - G3 > all groups (especially vs G2).  - Below detection limit (<23.2 ng/mL): more frequent in G1, less in G3.  4. Correlations  - citH3: negative with LA velocity and fractional shortening.  - citH3: stronger with LA/Ao, weak with neutrophils.  - cfDNA vs citH3: no direct correlation; moderate when grouped by bp.  5. NETs (Neutrophil Extracellular Traps)  - Higher in G2 and G3.  - Detectable citH3: 40% (G2), 80% (G3).  - Associated with thrombotic risk factors. | Li et al. (2023)*  DOI: 10.1177/1098612X211044986 |
|  |  |  | citH3 | | | | | | | | | (G1) 2380 ± 1254 ng/ml  (G2) 4101 ± 2350 ng/ml  (G3) 8618 ± 3978 ng/ml | | | | | | | | | | | | | | | |  |  |
| 2023  Research | - 80 felines with phenomenon of thin and hyperkinetic myocardial segments in cats with HCM.  - Age 6.6 years (4.5-8.7) (pre) and 9.1 years (7.6-10.6) (post).  - Mostly non-pedigree cats. | HCM | cTnI (n = 40) | | | | | | | | | 1.4 [0.07-180] | | | | | | | | | | | | | | | | - There was wide variation in the magnitude of myocardial injury among the cats studied. The authors did not assess cTnI longitudinally, reporting only the median value. | Novo Matos et al. (2023)  DOI: 10.1016/j.jvc.2023.02.002 |
| 2023  Research | - 80 felines, 3 groups  (G1) Healthy (n=25); 5 (2-8) years  (G2) HCM B (n=34); 6.8 (5.68-8.6) years  (G3) HCM C (n=21); 7.79 ± 3.63 years  - 53 neutered males and 27 spayed females. | HCM stage B and C | NT-proBNP (in 24 G1, 18 G2 and 19 G3), cTnI (in 21 G1, 18 G2 and 18 G3) and galectin-3 (all cats). | | | | | | | | | | | | | | | | | | | | | | | | | - It was observed that animals in G2 and G3 had higher Gal-3 levels compared to G1, with no differences between G2 and G3. -NT-proBNP tends to increase with disease severity. -cTnI was higher in group G3. - Gal-3 has moderate ability to differentiate animals with HCM stages. - Gal-3 concentrations >201 pg/mL show 81% sensitivity and 88% specificity for distinguishing healthy cats from stage C HCM. - Regarding NT-proBNP and cTnI, these are valid for differentiating stage B and C HCM from healthy cats, and also between each other. - Across all animals, Gal-3 shows a positive correlation with NT-proBNP, cTnI, urea, creatinine, and SDMA. A weak association was observed between Gal-3, urea, creatinine, left atrial dimension, and LA/Ao in linear regression. - Considering G2 and G3, there was a significant correlation of Gal-3 with left atrial dimension and LA/Ao.  - In particular, in G2, a strong correlation with extracellular volume fraction was observed.  - Gal-3 may be a potential biomarker in feline HCM. | Stack et al. (2023)*  DOI: 10.1016/j.jvc.2023.06.003 |
| 2023  Research | - Establishment of a colony of cats with HCM positive for the A31P MYBPC3 mutation by breeding a heterozygous male with females to obtain homozygous animals. - Serial follow-up from kittens to adults | HCM | NT-proBNP and cTnI | | | | | | | | | | | | | | | | | | | | | | | | | - NT-proBNP was associated with HCM, genetics, and obstruction. - Homozygous cats showed elevated NT-proBNP concentrations. cTnI did not differ between affected and healthy animals. - A cutoff of 100 pM was used, with many homozygous animals exceeding 200 pM. | Stern et al., (2023)*  DOI: 10.1038/s41598-023-36932-5 |
| 2023  Research | - 52 felines from surveys applied, 2 groups  (G1) High-pulse diets (n=21); 5.2 (3.3–7.9) years  (G2) Low-pulse diets (n=31); 4.3 (3.0–9.2) years  - Dietary consumption ranged from 6 months to 10 years, with a median duration of 2 years.  -Various breeds  (G1 and G2) Mostly NF and NM. | HCM | *Taurine – plasma (nmol/mL)* | | | | | | | | | (G1) 146 (123–184)  (G2) 137 (109–167) | | | | | | | | | | | | | | | | - No significant differences were identified between diet groups for NT-proBNP, cTnI, or plasma or whole-blood taurine concentrations. - There was no significant relationship between NT-proBNP, cTnI, and taurine among the diet groups.  - In G1, 71% of cats had values within the laboratory reference range (300–600 nmol/mL); the remainder were in the range considered outside the risk of deficiency. - In G2, 55% of cats had values within the laboratory reference range (300–600 nmol/mL); 3% were below, and the remainder were in the range considered outside the risk of deficiency.  - The authors noted that the lack of significant results was expected due to the minimal nutritional levels present in commercial cat diets. | Karp et al. (2023)*  DOI: 10.1177/1098612X231154859 |
|  |  |  | *Taurine – whole blood (nmol/mL)* | | | | | | | | | (G1) 348 (273–392)  (G2) 307 (250–341) | | | | | | | | | | | | | | | |  |  |
|  |  |  | *NT-proBNP (pmol/L)* | | | | | | | | | (G1) 24 (24–39)  (G2) 33 (24–46) | | | | | | | | | | | | | | | |  |  |
|  |  |  | *High-sensitivity cTnI (ng/mL)* | | | | | | | | | (G1) 0.026 (0.010–0.036)  (G2) 0.018 (0.000–0.052) | | | | | | | | | | | | | | | |  |  |
| 2024  Research | - 18 hearts – formalin-fixed, paraffin embedded, 3 groups  (G1) HCM I (primary origin) (n=10)  (G2) HCM II (secondary origin) (n=4)  (G3) Control (n=4) | HCM | - RNA expression and microRNAs - sarcomeric genes (TNNT2, TNNI3, MYH7, MYBPC3, TPM1 and ACTC1) | | | | | | | | | | | | | | | | | | | | | | | | | - There was increased expression of all six genes in cats with primary HCM compared to the control group.  - Comparing primary and secondary HCM, the primary form showed increased expression of all sarcomeric genes, except for TNNI3 and ACTC1.  - Expression of miR-370-3p, which was 4x higher in secondary HCM – it may be a possible tissue biomarker for discerning healthy cases of primary/secondary HCM.  - MicroRNAs miR-122-5p, miR-338-3p, miR-484, miR-30a-5p, miR-92b-3p, and miR-375, and miR-370-3p may be possible candidates for feline HCM. | Guelfi et al. (2024)  DOI: 10.1111/iep.12514 |
| 2024  Research | - 42 felines, 3 groups  (G1) Homozygous wild type (n = 17)  (G2) Homozygous *MYBPC3* gene mutation (n = 14)  (G3) Heterozygous *MYBPC3* gene mutation (n = 11).  -Various breeds | Healthy, but with MYBPC3 sarcomeric gene |  | | | | | | | | | *Serum galectin-3 (Gal-3)* | | | | | | | | | | | *Titin*  Neg (OD < 0.15)  Pos (OD ≥ 0.15) | | | | | - Gal-3 levels >250 pg/mL may predispose to the hypertrophy phenotype. However, only FS and LVPW were signif­icant.  - No differences in Gal-3 concentrations between groups.  - Titin may be a potential modulator of feline cardiac hypertrophy. | Demmekul et al. (2024)  DOI:  10.14202/vetworld.2024.2407-2416 |
|  |  |  | Overall | | | | | | | | | 204.5 ± 32.59 | | | | | | | | | | | Negative 34  Positive 8 | | | | |  |  |
|  |  |  | G1 | | | | | | | | | 229.39 ± 70.10 | | | | | | | | | | | Negative 14  Positive 3 | | | | |  |  |
|  |  |  | G2 | | | | | | | | | 188.11 ± 29.06 | | | | | | | | | | | Negative 12  Positive 2 | | | | |  |  |
|  |  |  | G3 | | | | | | | | | 259.23 ± 89.44 | | | | | | | | | | | Negative 8  Positive 3 | | | | |  |  |
|  |  |  | Without LVH | | | | | | | | | 243.28 ± 40.34 | | | | | | | | | | | Negative 26  Positive - | | | | |  |  |
|  |  |  | With LVH | | | | | | | | | 125.51 ± 40.09 | | | | | | | | | | | Negative 8  Positive 8 | | | | |  |  |
| 2024  Research | - 354 felines, but only 161 have cardiac disease (n=81 HCM).  - HCM felines: 7.00 (3.00–10.00) years, 23M, 26 NM, 17F, SF  - Various breeds | HCM | NT-proBNP | | | | | | Normal | | | | | | | | | | | | | Cardiac diseases | | | | | | -Cats with HCM had higher body weight. - Heart sounds, tachycardia (>200 bpm), NT-proBNP, pulmonary sounds, vertebral heart size (VHS) >8, and atrial dilation are potential indicators of cardiac disease. A respiratory rate >80 breaths/min was associated only with cardiopathies. - A predictive formula for HCM can be used, with Y_1_ = −3.637 + 2.448 (LA size) + 2.683 (murmur) + 1.274 (gallop), which showed an AUC of 0.899. When applied, it demonstrated useful sensitivity and specificity for ruling out the disease, as it confirmed only 41% of cases. | Tantitamtaworn et al. (2024)  DOI: 10.5455/OVJ.2024.v14.i9.29 |
|  |  |  | Normal  (*n* = 28) | | | | | | 25 | | | | | | | | | | | | | 3 | | | | | |  |  |
|  |  |  | Abnormal  (*n* = 72) | | | | | | 37 | | | | | | | | | | | | | 35 | | | | | |  |  |
| 2024  Research | - 20 felines  -11 felines had weight loss. -Median age was 7.4 years.  - Mostly neutered males. | Possible HCM | Before weight reduction (n=7) | | | | | | | | | | | | | Hs-cTnI: 0.007 (0.004–0.040)  NT-proBNP: 24.0 (23.9–31.0) | | | | | | | | | | | | - No changes were observed in the serum profiles of the evaluated biomarkers, even after weight loss. | Partington et al. (2024)*  DOI: 10.1186/s12917-024-04011-0 |
|  |  |  | After weight reduction (n=7) | | | | | | | | | | | | | Hs-cTnI: 0.005 (0.004–0.011)  NT-proBNP: 23.9 (23.9–75.0) | | | | | | | | | | | |  |  |
| 2024  Case Reports | - 2 cases of HCM  Case 1. Feline, 11-year-old, male neutered and Domestic Shorthair with syncope episodes during rest or after mild exercise.  Case 2. Feline, 10-year-old, male neutered and Domestic Shorthair with lethargy, apathy, and collapses in a 24-hour period. | Cases 1 and 2 - HCM ACVIM stage not described – LV hypertrophy > 7 mm. | Case 1. CTnI 2.1 ng/mL (VetScan iStat) | | | | | | | | | | | | | | | | | | | | | | | | | - There was an elevation of cTnI in both cases, but no serial measurements were performed to monitor injury.  - The authors also highlight that the biomarker, when associated with the electrocardiogram, allows for a better assessment of myocardial ischemia in felines, especially those with HCM. | Seo et al. (2024)  DOI: 10.1016/j.jvc.2024.05.003 |
|  |  |  | Case 2. cTnI 1.21 ng/mL (VetScan iStat) | | | | | | | | | | | | | | | | | | | | | | | | |  |  |
| 2024  Research | - 22 felines, 3 groups  (G1) Healthy (n=8); 8.8 ± 4.6 years  (G2) Subclinical HCM (n=8); 6.9 ± 3.1 years  (G3) HCM + CHF (n=6); 8.0 ± 4.6 years  - (G1 and G2) 6M/2F; (G3) 3F/3M  - Various breeds | (G2) B1/B2  (G3) C/D | *RNA*  *IGFBP -2** | | | | | G1/G2: higher gene expression compared with G3  G2 > G1 serum IGFBP-2  G3: serum IGFBP-2 gene expression was below detectable. | | | | | | | | | | | | | | | | | | | | - IGFBP-2, WNT5A, and IL-18 may be useful in feline HCM. | Chong et al. (2024)  DOI: 10.1016/j.rvsc.2024.105430 |
|  |  |  | *RNA IL-18** | | | | | G3 > G1 and G2 | | | | | | | | | | | | | | | | | | | |  |  |
|  |  |  | *RNA WNT5A** | | | | | G2 and G3 > G1 | | | | | | | | | | | | | | | | | | | |  |  |
|  |  |  | *RNA PYBG* | | | | | Not differences | | | | | | | | | | | | | | | | | | | |  |  |
|  |  |  | *Serum*  *IGFBP-2 \|*  *IL-18* | | | | | Not differences | | | | | | | | | | | | | | | | | | | |  |  |
| 2024  Research | - 29 felines, 2 groups  -(G1) Healthy cats; median 2.5 years; 60% male  -(G2) HCM cats with symptoms; median 2 years; 54.3% male | HCM | D-dimer (ng/ml) | | | | | | | | | (G1) 208.54 ± 10.92  (G2) 372.19 ± 58.28 | | | | | | | | | | | | | | | | - There was an elevation of D-dimer levels in cats from group G2.  - Prothrombin time decreased in G2 cats compared with G1; however, values remained within the reference range for both groups.  - Positive regulation of integrin alpha M subunit (ITGAM), elongin B (ELOB), and fibrillin 2 (FBN2) was observed.  - Negative regulation of zinc finger protein 316 (ZNF316) and ectonucleoside triphosphate diphosphohydrolase 8 (ENTPD8) was observed.  - Protein expression in cats may be considered a potential biomarker, but further studies with more homogeneous populations are still needed. | Jiwaganont et al. (2024)  DOI: 10.1186/s12917-024-04170-0 |
|  |  |  | Prothrombin time (seconds) | | | | | | | | | (G1) 11.08 ± 0.23  (G2) 9.8 ± 0.15 | | | | | | | | | | | | | | | |  |  |
|  |  |  | Protein regulations (upregulation and downregulation) | | | | | | | | | | | | | | | | | | | | | | | | |  |  |
| 2025  Research | - 62 felines, 3 groups  (G1) Control (n=35)  (G2) HCM B1+B2 (n=13)  (G3) HCM C + D (n=14)  - Mean age 3.9 years in G1 group and 8.2 years in HCM group.  - Most cats in G1 were 0–5 years old, while those with HCM were 6–12 years old.  - Most cats: male neutered. | HCM B1 to D |  | | | | IGF-1 | | | | | | | | | 26S PSM | | | | | | | | | 3-MH | | | - There is detection of IGF-1, 26S PSM, and 3-MH in both healthy cats and those with HCM. Higher concentrations of IGF-1 and 3-MH were found in the HCM group, with no difference observed for 26S PSM.  - IGF-1: Higher medians were observed in HCM cats with and without atrial dilation. Differences in IGF-1 were found between HCM cats (with and without increased LA/Ao) and healthy cats, as well as between stages B1+B2 and C+D; no internal differences were detected.  - 26S PSM: No significant differences were observed.  - 3-MH: Significant differences were found between healthy cats and HCM cats with and without atrial enlargement, LA/Ao (with/without), and between stages B1+B2 and C+D (higher in the HCM group).  - No correlations were found between these biomarkers and age, weight, morphological, or clinical variables.  - A potential interaction may be suggested between HCM and metabolic proteins. | Neumann et al. (2025)  DOI: 10.3390/  ani15101437 |
|  |  |  | Control | | | | 23.0 [10.5; 77.6] | | | | | | | | | 0.61 [0.18; 3.58] | | | | | | | | | 128 [25.1; 345] | | |  |  |
|  |  |  | HCM | | | | 46.2 [27.8; 59.9] | | | | | | | | | 0.55 [0.20; 3.49] | | | | | | | | | 373 [229; 1141] | | |  |  |
|  |  |  | HCM B1 + B2 | | | | 50.8 [27.8; 58.8] | | | | | | | | | 0.42 [0.22; 2.03] | | | | | | | | | 370 [183; 563] | | |  |  |
|  |  |  | HCM C + D | | | | 41.2 [32.6; 59.9] | | | | | | | | | 0.56 [0.20; 3.49] | | | | | | | | | 675 [112; 1141] | | |  |  |
| 2025  Research | - 12 felines, 2 groups  (G1) Healthy  (G2) HCM  - microRNA analysis  -Domestic mixed breed or Norwegian Forest cat.  -Variable ages (2.43 -13.8 years)  - Only 4 female cats. | HCM ACVIM stage not described | miRNAs | | | | | | | | | | | | | | | | | | | | | | | | | - 459 fully processed miRNAs were identified, including 40 potential new feline miRNAs. - Approximately 85.3% of the miRNAs showed sequence similarity with human miRNAs. - Some exhibited differences in expression between breeds. - In Norwegian cats, miR-204-5p showed a possible association with preclinical HCM. - Breed should be taken into account when evaluating miRNA. | Ohlsson et al. (2025)  DOI: 10.1038/s41598-025-09478-x |
| 2025  Research | -Feline, male, 8-year-old  - Domestic longhair  - ATE clinical signs (e.g. sudden forelimb paresis and vocalization).  - Heart murmur + CHF | HCM end-stage | Before ATE/CHF management | | | | | | | | | | | | | | | Before ATE/CHF management | | | | | | | | | | - The findings showed a reduction in cTnI concentrations after therapeutic management of CHF/ATE secondary to HCM, with the increase in the biomarker being attributed by the authors to a condition of acute myocardial injury. | Saponaro et al. (2025)  DOI: 10.1002/vrc2.1073 |
|  |  |  | cTnI  3.95 ng/mL (reference < 0.06) | | | | | | | | | | | | | | | cTnI  0.27 ng/mL (reference < 0.06) | | | | | | | | | |  |  |
| 2025  Research | - 46 felines, 2 groups  (G1) HCM (n=13); 3.4 (1.7 - 7.1) years, 6M/7F.  (G20 HOCM (n=33); 2.7 (1.4 - 5.7) years, 23M/10F. | HCM and HOCM  ACVIM stage not described |  | | | | | | HCM | | | | | | | | | | | HOCM | | | | | | | | - It was observed that cTnI was higher in animals with obstructive HCM. - In 31% of cases, non-obstructive HCM showed elevated cTnI, while in 73% of cases with obstructive HCM. - There are significant correlations between cTnI and the maximum outflow tract velocity.  - cTnI was associated with obstructive HCM. | Satomi et al. (2025)*  DOI: 10.3390/  ani15091313 |
|  |  |  | cTnI* | | | | | | 0.069 (0.029 - 0.156) | | | | | | | | | | | 0.311 (0.066 - 0.500) | | | | | | | |  |  |
| 2025  Research | - Left atrioventricular tissue samples from healthy (n=8) and HCM (n=12) felines. | HCM  ACVIM stage not described | MicroRNAs miR-132, miR-124-3p, miR-122-5p, miR-185-3p e miR-153-3p e genes/moléculas TNFα, IL1β, TGFβ, FGF23 e HIF1α. | | | | | | | | | | | | | | | | | | | | | | | | | - A total of 215 genes and approximately 15 microRNAs with atrioventricular expression were observed in cats with HCM. - Left ventricular region: upstream regulation expression of miR-122-5p, miR-124-3p, and miR-132, and downregulation of miR-122-5p. - Left atrial region: upstream regulation expression of miR-185-3p and miR-153-3p. - Interactions were found associated with pathways such as HIF1α, TGFβ, and hypertrophy. - Activation of pathways including TNFα, IL1β, TGFβ, and FGF23 was observed. - Macrophage activation pathways were more prominently observed in the left atrium. | Joshua et al. (2025)  DOI: 10.3390/ijms26146764 |
| 2025  Research | - 78 felines, 3 groups  (G1) Healthy (n=16); 6.5 (±1.9) years; 10M/6F.  (G2) Felines with primary cardiomyopathy (n=37); 6.2 (±0.8) years; 23M/14F; primary HCM (24/37 cats)  (G3) Felines with secondary cardiomyopathy (n=25); 8.2 (±1.1) years; 21M/4F.  - Various breeds. | HCM in majority cases  ACVIM stage not described | cTnI (ng/mL) | | | | | | | | | | | | G1: 0.13 ± 0.01 G2: 0.30 ± 0.02 G3: 0.24 ± 0.01 | | | | | | | | | | | | | - Significant increase in AST in G2 compared to G1 and G3. - G2 and G3 showed significant increases in cTnI, CK-MB, CK, and CK-RI. - cTnI - G2 > G3 > G1; in G2 it was 1.5x higher than in G3. - G2 had increased NT-proBNP and MR-proADM compared to G1; NT-proBNP in G3 > G1. Gal-3 was elevated in G2 and G3 > G1. - Strong positive correlation between NT-proBNP and cTnI; moderate correlations between NT-proBNP and CK-MB, CK-RI, and creatinine. - Moderate positive correlations were observed between cTnI and CK, CK-MB, and AST; weak correlations with CK-RI and creatinine. -CK-MB:moderate positive correlations with creatinine and CK-RI. CK showed moderate positive correlations with AST, MR-proADM, and Gal-3; AST/ALT correlated moderately with MR-proADM. - cTnI showed 60.7% sensitivity and 82.4% specificity for differentiating healthy from diseased cats (cutoff 0.18 ng/mL). CK-MB had 70% sensitivity and 64.7% specificity: less specific than cTnI.  - CK-RI had 50.8% sensitivity and 100% specificity (cutoff 2.84%). NT-proBNP had 72% sensitivity and 88% specificity (cutoff 213 pmol/L). MR-proADM had 42.9% sensitivity and 87.5% specificity. Gal-3 had 26.2% sensitivity and 58.8% specificity. - Overall, NT-proBNP, cTnI, CK-MB, and CK-RI provided the greatest benefit, with cTnI particularly useful for distinguishing primary and secondary cardiomyopathies, and can be used when echocardiography is unavailable. | Abdelhaleem et al. (2025)  DOI: 10.1016/j.tvjl.2025.106441 |
|  |  |  | CK-MB (ng/mL) | | | | | | | | | | | | G1: 5.56 ± 0.19 G2: 11.68 ± 0.66 G3: 12.65 ± 0.89 | | | | | | | | | | | | |  |  |
|  |  |  | CK (IU/L) | | | | | | | | | | | | G1: 247.46 ± 12.44 G2: 494.30 ± 37.85 G3: 415.58 ± 54.37 | | | | | | | | | | | | |  |  |
|  |  |  | CK-RI (%) | | | | | | | | | | | | G1: 1.27 ± 0.18 G2: 2.79 ± 0.32 G3: 3.62 ± 0.38 | | | | | | | | | | | | |  |  |
|  |  |  | NT-proBNP (pmol/L) | | | | | | | | | | | | G1: 201.77 ± 1.01 G2: 379.50 ± 15.37 G3: 361.63 ± 10.72 | | | | | | | | | | | | |  |  |
|  |  |  | MR-proADM (pg/mL) | | | | | | | | | | | | G1: 13.93 ± 0.45 G2: 24.22 ± 1.59 G3: 13.70 ± 0.31 | | | | | | | | | | | | |  |  |
|  |  |  | Gal-3 (ng/mL) | | | | | | | | | | | | G1: 20.43 ± 2.19 G2: 32.30 ± 1.12 G3: 26.50 ± 1.46 | | | | | | | | | | | | |  |  |
| 2026  Research | 202 felines, 5 groups  - Various breeds  (G1) All cats (n=202), 84.5 (31-130) months, ~72%M  (G2) Cardiac disease (n=123), 75 (28-128) months, ~76%  (G3) Normal (n=79), 98 (31-132) months, ~66%  (G4) HCM B2 (n=24), 72.5 (22-140) months, 75%  (G5) Normal + HCM B1 (n=178), 87 (31-130) months, ~71% | Some cats HCM  ACVIM B1 and B2 |  | (G1) | | | | | | | (G2) | | | | (G3) | | | | | | (G4) | | | | | | (G5) | - NT-proBNP  Higher concentrations in cats with cardiac disease than in healthy cats.  Concentrations were highest in cats with stage B2 disease.  A cutoff of ≥109 pmol/L independently predicted cardiac disease.  Diagnostic accuracy improved when combined with clinical variables.  - hs-cTnI  Higher concentrations in cats with cardiac disease than in healthy cats.  Concentrations were highest in cats with stage B2 disease.  Failed to independently predict cardiac disease and was excluded from the final screening model. | Carter et al. (2026)*  DOI: 10.1093/jvimsj/aalaf037 |
|  |  |  | NT-proBNP (pmol/L) | 86 (45-280) | | | | | | | 136 (54-467) | | | | 57 (37-95) | | | | | | 515.5 (131-901) | | | | | | 77  (43-194) |  |  |
|  |  |  | hs-cTnI (ng/mL) | 0.069 (0.022-0.178) | | | | | | | 0.086 (0.035-0.183) | | | | 0.027 (0.009-0.121) | | | | | | 0.138 (0.096-0.368) | | | | | | 0.057 (0.020-0.153) |  |  |
| 2026  Research | Serum samples – 106 client-owned  6 groups, 1-14 years  (G1) Healthy (n=51), 3.0 (1.0-12.0) years, 29M/22F  (G2) HCM B1 (n=25), 7.0 (1.0-14.0) years, 16M/9F  (G3) HCM B2 (n=7), 7.0 (4.0-9.0) years, 6M/1F  (G4) Cardiomyopathy C (n=7), 3.0 (0.4-14.0) years, 6M/1F  (G5) Congenital (n=8), 1.5 (0.8-9.0) years, 4M/4F  (G6) Transient myocardial thickening/myocarditis (n=8), 6.5 (0.6-14.0) years, 5M/3F | Some HCM cats  ACVIM B1, B2 and C | cTnI (pg/mL) | | | | | | | | | | | | | | | | | | | | | | | | | - The cTnI assay demonstrated adequate precision, reproducibility, and accuracy for measuring serum cTnI concentrations in cats, with a limit of quantification of 8 pg/mL.  - The reference interval for healthy cats was <8–190 pg/mL. | Lidbury et al. (2026)  DOI: 10.1371/journal.pone.0346522 |
|  |  |  | (G1) | | | | | | | | | | | | 26 (<8-193) | | | | | | | | | | | | |  |  |
|  |  |  | (G2) | | | | | | | | | | | | 94 (<8-415) | | | | | | | | | | | | |  |  |
|  |  |  | (G3) | | | | | | | | | | | | 268 (29 –2,270) | | | | | | | | | | | | |  |  |
|  |  |  | (G4) | | | | | | | | | | | | 369 (26 –1,073) | | | | | | | | | | | | |  |  |
|  |  |  | (G5) | | | | | | | | | | | | 75 (18 –10,602) | | | | | | | | | | | | |  |  |
|  |  |  | (G6) | | | | | | | | | | | | 4,532 (510−25,001) | | | | | | | | | | | | |  |  |
| 2026  Research | - 86 felines, 3 groups, 8.9 ± 4.6 years, 3M/63NM/20NF  (G1) HOCM (n=31), 7.7 ± 4.8 years, 3M/22NM/0F/6NF  (G2) CHF (n=30), 9.4 ± 5.0 years, 0M/25NM/0F/5NF  (G3) ATE (n=25), 9.6 ± 3.9 years, 0M/16NM/0F/9NF  - Most breeds - domestic short- and long-hair | HCM cats  ACVIM stage not described | All cats | | | | 945 (19–1501) | | | | | | | | | | | | | | | | | | | | | - Cats in G2 and G3 had higher NT-proBNP concentrations than those in G1, with no significant difference between G2 and G3.  - An NT-proBNP cutoff value of 491 pmol/L yielded a sensitivity of 96.0% and a specificity of 93.5%.  - Cats with NT-proBNP concentrations >491 pmol/L were more likely to present with a gallop rhythm and CHF, as well as higher values of LVIDs, IVSd, LVWs, LVWdmax, and LA/Ao, but lower LAA velocities and FS%.  - NT-proBNP concentrations >491 pmol/L were also associated with an increased likelihood of thrombus formation and SEC.  - NT-proBNP failed to differentiate between cats with CHF and those with ATE. | Oranges et al. (2026)  DOI: 10.3390/ani16020157 |
|  |  |  | NT-proBNP (pmol/L) | | | | (G1) | | | | | | | | (G2) | | | | | | | | | (G3) | | | |  |  |
|  |  |  |  |  |  |  | 127  (19–913) | | | | | | | | 1484  (407–1501) | | | | | | | | | 1500  (402–1501) | | | |  |  |
| 2026  research | 11 felines, 02 groups  (G1) Standard-dose carvedilol, 2.0 (1.2–3.9) years  (G2) High-dose carvedilol, 2.4 (1.5–3.9) years  - 11 males, 4 females | HCM stage B1 obstructive | *cTnI* | | | | | | | | | | | | | | | | | | | | | | | | | - There was a reduction in HR with high-dose carvedilol treatment compared with low dose. Blood pressure values remained unchanged.  - Standard-dose carvedilol reduced the magnitude of clinical signs in some cats.  - cTnI levels showed a significant difference with high-dose treatment.  - IVSd, LVPWd, and maximal wall thickness decreased after carvedilol treatment (both low and high dose). In some cats, high dose reduced LVOTVrest and LVOTVexcited.  - Longitudinal endocardial and epicardial strain improved with both low and high doses of carvedilol.  - Positive correlations were observed between cTnI and LVOTVrest/LVOTVexcited, with LVOTVexcited showing the strongest correlation with cTnI. A negative correlation was found between cTnI and longitudinal strain.  - There is a dose-dependent effect of carvedilol in reducing LVOTO in cats with obstructive HCM B1.  - High-dose carvedilol improves cTnI concentrations and clinical signs. | Satomi et al. (2026)  DOI: 10.1177/1098612X261433060 |
|  |  |  | *Case* | | | | *Before (ng/ml)* | | | | | | | | *SD (ng/ml)* | | | | | | | | | *HD (ng/ml)* | | | |  |  |
|  |  |  | 1 | | | | 0.356 | | | | | | | | 0.125 | | | | | | | | | 0.018 | | | |  |  |
|  |  |  | 2 | | | | ND | | | | | | | | 0.196 | | | | | | | | | 0.007 | | | |  |  |
|  |  |  | 3 | | | | 1.739 | | | | | | | | 0.558 | | | | | | | | | 0.453 | | | |  |  |
|  |  |  | 4 | | | | 0.583 | | | | | | | | 0.125 | | | | | | | | | 0.010 | | | |  |  |
|  |  |  | 5 | | | | 0.278 | | | | | | | | 0.094 | | | | | | | | | 0.161 | | | |  |  |
|  |  |  | 6 | | | | 0.311 | | | | | | | | 0.192 | | | | | | | | | 0.155 | | | |  |  |
|  |  |  | 7 | | | | 6.38 | | | | | | | | 1.153 | | | | | | | | | 0.182 | | | |  |  |
|  |  |  | 8 | | | | 0.032 | | | | | | | | ND | | | | | | | | | <0.003 | | | |  |  |
|  |  |  | 9 | | | | 0.662 | | | | | | | | 0.196 | | | | | | | | | 0.011 | | | |  |  |
|  |  |  | 10 | | | | 0.133 | | | | | | | | 0.096 | | | | | | | | | 0.009 | | | |  |  |
|  |  |  | 11 | | | | 0.070 | | | | | | | | ND | | | | | | | | | 0.051 | | | |  |  |

Consider: ACE - angiotensin-converting enzyme; ADRB1 - cardiac beta-1 adrenergic receptor; AGP - alpha-1 acid glycoprotein; aPTT - activated partial thromboplastin time; ATE - arterial thromboembolism; B1/B2/C/D - ACVIM stages of hypertrophic cardiomyopathy; Ca^2+^ - calcium 2+; cfDNA - cell-free DNA; CHF - congestive heart failure; citH3 - citrullinated histone H3; CITP - C-terminal telopeptide of type I procollagen; CK - creatine kinase; CKD – chronic kidney disease; CK-MB - creatine kinase–MB isoenzyme; CK-RI - creatine kinase–RI isoform index (as defined by authors); cTnI - cardiac troponin I; cTnT - cardiac troponin T; CATE - cardiac thromboembolic events; DM – diabetes mellitus; DSH - domestic shorthair cat; EGCG - epigallocatechin 3-gallate; ELISA - enzyme-linked immunosorbent assay; FIP - feline infectious peritonitis; FS - fractional shortening; Gal-3 - galectin-3; HCM - hypertrophic cardiomyopathy; HF – heart failure; HOCM - hypertrophic obstructive cardiomyopathy; Hs-cTnI - high-sensitivity cardiac troponin I; IGF-1 - insulin-like growth factor 1; IGFBP-2 - insulin-like growth factor binding protein 2; IQR - interquartile range; IL - interleukin; IVSd - interventricular septal thickness in diastole; IVSs - interventricular septal thickness in systole; LA - left atrium; LA/Ao - left atrium-to-aorta ratio; LAE - left atrial enlargement; LV - left ventricle; LVFWd - left ventricular free wall thickness in diastole; LVH – left ventricular hypertrophy; LVOT - left ventricular outflow tract; MMP - matrix metalloproteinase; MST - median survival time; MR-proADM - mid-regional pro-adrenomedullin; MYBPC3 - myosin-binding protein C3 gene; ND – no data; NF - neutered female; NM - neutered male; NT-proBNP - N-terminal pro–B-type natriuretic peptide; OD - optical density; PIIINP - N-terminal of type III procollagen; POC/POCT - point-of-care test; RDW – red cell distribution width; SAA - serum amyloid A; SAM - systolic anterior motion (of the mitral valve); SDMA - symmetric dimethylarginine; sST2 - soluble suppression of tumorigenicity 2; TGF-β - transforming growth factor beta; TIMP - tissue inhibitor of metalloproteinases; TNFα - tumor necrosis factor alpha; VHS - vertebral heart size.

* Reported manufacturer-related funding, editorial roles, or potential conflicts of interest.
